# Multichannel microneedle dry electrode patches for minimally invasive transdermal recording of electrophysiological signals

**DOI:** 10.1038/s41378-024-00702-8

**Published:** 2024-05-31

**Authors:** Zhengjie Liu, Xingyuan Xu, Shuang Huang, Xinshuo Huang, Zhibo Liu, Chuanjie Yao, Mengyi He, Jiayi Chen, Hui-jiuan Chen, Jing Liu, Xi Xie

**Affiliations:** 1 State Key Laboratory of Optoelectronic Materials and Technologies, School of Electronics and Information Technology, Guangdong Province Key Laboratory of Display Material and Technology, Sun Yat-Sen University, Guangzhou, China; 2 School of Biomedical Engineering, Sun Yat-Sen University, Guangzhou, China; 3 The First Affiliated Hospital of Sun Yat-Sen University, Guangzhou, China

**Keywords:** Electrical and electronic engineering, Sensors

## Abstract

The collection of multiple-channel electrophysiological signals enables a comprehensive understanding of the spatial distribution and temporal features of electrophysiological activities. This approach can help to distinguish the traits and patterns of different ailments to enhance diagnostic accuracy. Microneedle array electrodes, which can penetrate skin without pain, can lessen the impedance between the electrodes and skin; however, current microneedle methods are limited to single channels and cannot achieve multichannel collection in small areas. Here, a multichannel (32 channels) microneedle dry electrode patch device was developed via a dimensionality reduction fabrication and integration approach and supported by a self-developed circuit system to record weak electrophysiological signals, including electroencephalography (EEG), electrocardiogram (ECG), and electromyography (EMG) signals. The microneedles reduced the electrode–skin contact impedance by penetrating the nonconducting stratum corneum in a painless way. The multichannel microneedle array (MMA) enabled painless transdermal recording of multichannel electrophysiological signals from the subcutaneous space, with high temporal and spatial resolution, reaching the level of a single microneedle in terms of signal precision. The MMA demonstrated the detection of the spatial distribution of ECG, EMG and EEG signals in live rabbit models, and the microneedle electrode (MNE) achieved better signal quality in the transcutaneous detection of EEG signals than did the conventional flat dry electrode array. This work offers a promising opportunity to develop advanced tools for neural interface technology and electrophysiological recording.

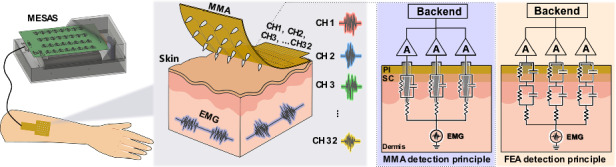

## Introduction

The detection of electrophysiological signals, including electroencephalogram (EEG), electrocardiogram (ECG), and electromyogram (EMG) signals, is important for studying the generation and transmission of electrical signals in cells and tissues^1,2^. It plays a crucial role in guiding the diagnosis and monitoring of diseases related to bioelectric signals^3^. For instance, EMG signals can reflect the activity and functional status of muscles, making them invaluable in clinical diagnostics and rehabilitation training within the neuromuscular system^4,5^. By examining EMG signals from affected areas, parameters such as denervation potentials and nerve conduction velocity can be obtained, aiding in assessing the extent of a nerve injury^6,7^. Additionally, in rehabilitation training, monitoring EMG signals provides quantitative information about muscle electromyographic output, assisting healthcare professionals in developing precise treatment and rehabilitation plans^8^. Moreover, real-time brain activity data can be recorded from EEG signals, providing valuable information for neuroscience research, clinical diagnosis and treatment^9,10^. In epilepsy research, analyzing the characteristics of different types of seizures in EEG recordings helps doctors determine the specific type of epilepsy^11,12^.

With the advancement of microfabrication techniques for electrodes at the micro- and nanoscale, significant progress has been made in multichannel electrophysiological signal acquisition technology^13^. Through processes such as photolithography and metal deposition, multichannel microelectrode patterns can be fabricated on a planar substrate. This allows for the collection of physiological electrical signals from various points in different tissue regions. The acquisition of multichannel physiological signals provides information on the spatiotemporal distribution of tissue electrophysiology, facilitating the accurate reconstruction of organ electrophysiological information^14^. For example, Lee et al. developed a graphene-based multichannel electrode array for recording electrocorticography (ECoG) signals, enabling the detection of neural responses to auditory and somatosensory stimuli on the cortical surface^15^. In the case of EMG signals, multichannel EMG signal acquisition reveals the transmission process of signals driving muscle contractions^9^. This is highly beneficial for basic research on neuromuscular control and the clinical diagnosis of neuromuscular diseases. Single-electrode or few-electrode approaches cannot fully capture the nonuniformity of muscle activation, while multichannel acquisition aids in analyzing the activity information of individual muscle groups and multiple muscle groups^16^.

In the context of clinical applications and scientific research related to surface electrophysiology, there is an urgent demand for multichannel, low-noise, and highly interference-resistant surface electrophysiological signal acquisition electrodes to obtain more detailed temporal and spatial information^17^. Surface electrophysiological signals are obtained by placing noninvasive electrodes on the surface of the skin. The high impedance of the stratum corneum (SC) layer of the skin affects the collection of weak electrophysiological signals^18^. The delicate and soft texture of the skin can create air gaps when electrodes are attached to it, increasing the impedance between the electrodes and the skin. Wet electrodes, which use conductive gels, effectively reduce the contact impedance between the skin and electrodes, thereby improving the quality of the detected electrophysiological signals^19^. Dry electrodes, on the other hand, incorporate micro-nanostructures to improve electrophysiological signal acquisition^20^. In recent years, there has been progress in improving electrode performance by using novel materials. For example, Song et al. developed a biomimetic, breathable, and sweat-stable MXene electrode with low and stable electrode–skin interface impedance in various environments, enabling reliable long-term electrophysiological detection^21^. However, the fabrication of electrodes using advanced micro/nanomaterials is specialized and complex, and mass production remains a challenge. In recent years, microneedle-based transdermal technology has made promising progress in drug delivery and biochemical sensing^22–24^. Microneedles, with an effective length that only penetrates the SC without reaching nerves or blood vessels, offer painless transdermal delivery^25^. Microneedle arrays have made promising progress in fields such as painless vaccine delivery, insulin delivery for diabetes treatment and antihypertensive drug delivery for hypertension treatment. In addition, single-channel electrochemical sensors based on microneedle arrays have made positive progress in applications such as diabetes detection and reactive oxygen species sensing^26^. In terms of physiological signal recording, microneedles directly penetrate the SC, bypassing its high-impedance properties^27^. Moreover, after transdermal penetration, microneedle tips remain in contact with the skin’s electrolyte, reducing the resistance between the electrode and the tissue gap^28,29^. The painless transdermal nature of microneedles also lends itself to their application as a minimally invasive transdermal technology in wearable sensing devices^30^. For instance, Krieger et al. fabricated 3D-printed stainless steel microneedle arrays using metal laser sintering, improving the long-term sensing stability and eliminating the need for skin preparation^31^. Li et al. developed flexible microneedle array electrodes that demonstrated improved signal acquisition for ECG and EMG signals compared to commercial wet electrodes, as well as higher sensing quality in a sweat environment^32^. However, current microneedle methods are limited to single-channel or limited-channel real-time signal acquisition and cannot achieve multichannel collection in small areas. While 2D planar electrodes can be fabricated using micro-nanofabrication processes to create high-density microelectrode arrays, the fabrication of multichannel electrode arrays with 3D microneedles using micro-nanofabrication processes remains challenging.

**Fig. 1:**
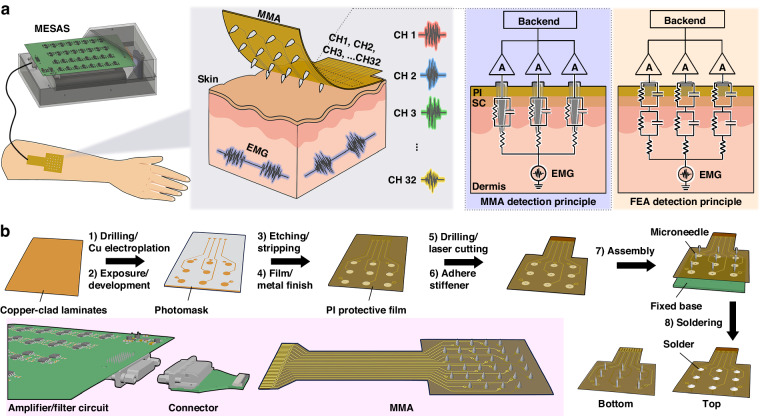
Schematic diagram and fabrication process of the MMA for EMG signal detection. **a** Schematic illustrating the use of the MMA sensor worn on the arm for real-time monitoring of EMG signals. A multichannel electrophysiological acquisition system (MESAS) was used to process the electrophysiological signals generated by the MMA, and the digital signals were ultimately transmitted to the computer for signal display. **b** Schematic diagram of the fabrication process of the MMA. A flexible printed circuit (FPC) process was utilized in which the copper foil substrate was initially cut into the desired pattern. Subsequently, holes were drilled and plated at the over-hole locations to achieve the desired configuration. Next, a photosensitive dry film was pressed onto the copper foil substrate and exposed, and a pattern of pad arrays, leads, etc., was formed through processing with a developing solution. The excess copper foil was etched away with an etching solution, and the dry film was peeled off to obtain the desired circuit pattern. The pads were chemically gold-plated to provide corrosion and oxidation resistance, and polyimide (PI) insulating material was employed to effectively insulate the leads. The prepared flexible circuit board was positioned on a rigid board (FR4 substrate), and the microneedles were inserted vertically. Finally, microneedles were soldered to the pads using solder, resulting in the final MMA sensor

In this study, a multichannel (32 channels) microneedle dry electrode patch device was developed using a dimensionality reduction fabrication and integration approach (Fig. 1a). This device allowed painless transdermal recording of electrophysiological signals from subcutaneous tissue, including EEG, EMG and ECG signals. First, this device achieved multichannel electrophysiological signal acquisition with high temporal and spatial resolution, reaching the level of a single microneedle in terms of signal accuracy. Second, it reduced the electrode–skin contact impedance by penetrating the nonconducting SC without contacting deeper tissues where nerves and blood vessels are present, thereby obtaining high-quality electrophysiological signals with relatively little tissue invasion. Third, the device exhibited high mechanical stability. The microneedle structure effectively secured the electrode to the skin, preventing signal instability caused by electrode movement. This work used electrical theoretical modeling, COMSOL mechanical simulation and in vitro transdermal impedance testing experiments to validate the beneficial effect of microneedle transdermal penetration on reducing electrode–skin impedance and increasing the amplitude of collected subcutaneous electrophysiological signals. In addition, a circuit system for the synchronous collection of weak electrophysiological signals from 32 channels was developed to support the operation of multichannel microneedle array (MMA) recording. Subsequently, experiments on live rabbits confirmed that MMA effectively collected subcutaneous ECG, EMG and EEG signals from rabbits, and the spatial distribution of the electrophysiological signals was recorded using multichannel microneedles. Furthermore, by integrating microneedle electrodes (MNEs) and flat electrodes (FEs) in the same device, it was confirmed that the MMA achieves better signal quality in the transcutaneous detection of EEG signals than does a conventional flat dry electrode array. The MMA developed in this study provides a promising strategy for improving the signal quality of dry electrode electrophysiological signal acquisition. The development of this technology not only has potential applications in the field of brain-computer interface technology but also provides a promising strategy for the advancement of neural signal monitoring techniques.

## Results and discussion

**Fig. 2:**
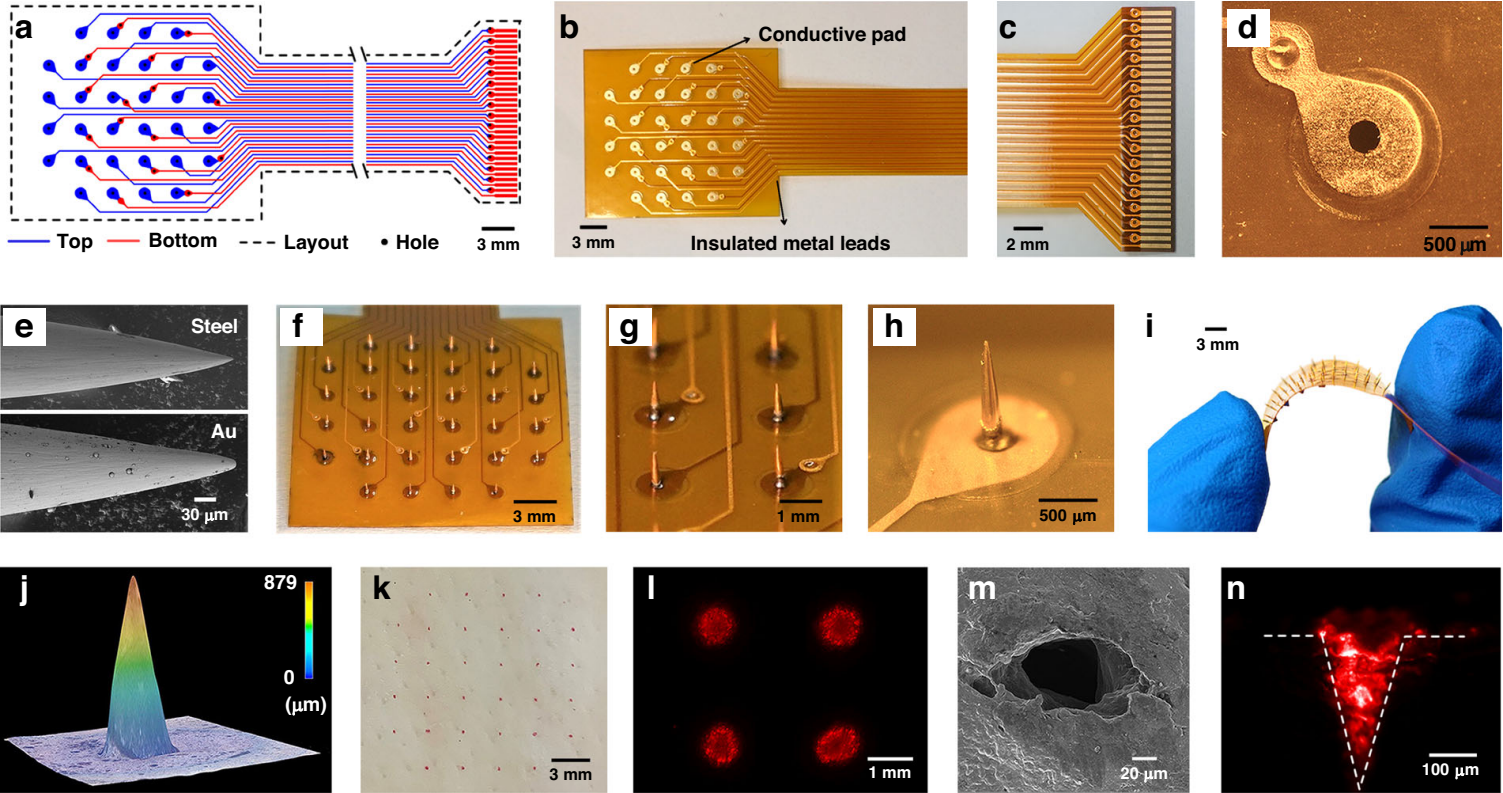
Fabrication and characterization of MMA. **a** The blue color indicates the top layer, the red color signifies the bottom layer, the black area indicates the drilling area, and the dotted line indicates the board frame in the flexible circuit board design layout of the MMA device. **b** Photograph of the flexible circuit board. **c** Photograph of the FPC interface. **d** Photograph of the exposed pads used for soldering the microneedles. **e** SEM images displaying the surface morphology of a microneedle before and after gold plating. **f** Photographs from the side of the MMA. **g** Enlarged photograph depicting the MMA with two microneedles spaced 3 mm apart. **h** Magnified optical microscope image displaying a conical microneedle tip with a height of approximately 800 μm. **i** Image of the MMA during bending showing that the microneedle tip remained perpendicular to the flexible substrate. **j** 3D reconstructed image depicting the morphology of the MMA. **k** Top view of porcine skin after microneedle penetration, with the indentation marked by red dye following MMA insertion. **l** Fluorescence images of the pig skin were captured after insertion by pressing the MMA. **m** SEM image of the pig skin pierced by the MMA. **n** Fluorescence images demonstrating the deposition of rhodamine B into the pig skin through MNE penetration

The fabrication procedure of the MMA is shown in Fig. 1b. A double-sided flexible circuit board (Fig. 2a) was designed with an array of 32 pads for assembly with microneedle tips. Each solder pad possessed a hole in the center with a 250 μm diameter that allowed the vertical insertion of the microneedles into the flexible circuit board. To reduce interference between electrode signals, the spacing between solder pads was at least 3 mm. To ensure that the leads maintained a specific separation during the wiring process to reduce mutual interference, adjacent wires were routed alternately on the top and bottom layers. All leads were insulated with a PI thin film and led out through an FPC interface (32 pins) at the other end. The flexible circuit board was prepared according to the following procedure. The copper foil substrate was cut according to the as-designed pattern, and holes were drilled at the via locations, followed by electroplating to create the necessary vias. The photoresist was laminated to the copper foil substrate and exposed. The development process created patterns such as solder pad arrays and leads. The excess copper foil was etched away using an etching solution, the dry film was removed, and the desired circuit pattern was obtained. The solder pads were chemically plated with gold to improve biocompatibility and oxidation resistance, and the leads were insulated with a PI layer. The as-prepared flexible circuit board, as shown in Fig. 2b, had most of its areas covered with PI film, with only the solder pads and the FPC interface exposed. The exposed solder pads and the surface of the FPC interface (Fig. 2c) were coated with gold to improve the connection reliability and conductivity. As shown in Fig. 2d, the exposed solder pads were 1 mm in size, and the holes drilled in their centers had a diameter of approximately 250 μm. This flexible circuit board served as the base substrate for the microneedle array.

Stainless steel needle tips with a diameter of 200 μm were then cut by a laser to produce needles approximately 1 cm in length. A gold layer was modified on the needle tips by electrochemical deposition to improve the biocompatibility. Scanning electron microscopy (SEM) was used to characterize the structure and surface morphology of the electrodes (Fig. 2e). After electroplating, the gold electrode surface exhibited a smooth appearance without noticeable cracks, indicating that the gold plating process achieved relatively uniform modification of the microneedle surfaces. The microneedle tips had a diameter of 0.1 μm, and this sharp needle-like structure facilitated efficient penetration of the skin surface. To solder the gold-plated microneedles vertically onto the flexible circuit board, a custom-made FR4 substrate with a thickness of approximately 800 μm was used (Fig. S1-1). The flexible circuit board was superimposed on the FR4 substrate. By adjusting the thickness of the FR4 substrate, the length of the microneedles protruding from the flexible circuit board could be adjusted. By ensuring that the holes on the flexible circuit board and the FR4 substrate were aligned, the microneedles were able to stand vertically when inserted into the microholes of the solder pads on the flexible circuit board. Next, an electric iron and stainless steel solder were used to connect the microneedles to the solder pads on the flexible circuit board, ensuring the formation of smooth solder joints to increase the reliability of the connection. Excess parts of the microneedles were trimmed off with diagonal pliers, and the remaining microneedles were soldered individually onto the flexible circuit board; this process was repeated until all 32 microneedles were successfully soldered.

Based on the design and fabrication processes described above, the resulting MMA device is shown in Fig. 2f. Each needle was oriented perpendicular to the substrate, and each needle tip had a conical shape. Since the base of each needle was connected to a solder pad, each microneedle corresponded to an independent electrical pathway on the flexible circuit board, ensuring that each microneedle had the ability to record individual electrophysiological signals. Each needle was exposed above this base (Fig. 2h), which consisted entirely of exposed metal. The areas below the needle tips were insulated. Thus, only the exposed needle tips above the base were conductive, which helped to reduce interference from other metal conductors at the base. When the device was inserted into the skin, the microneedle tips penetrated the skin tissue and received electrical signals from the tissue. The part in contact with the outermost layer of skin was the substrate base, which reduced signal interference from the skin surface, allowing the recorded electrophysiological signals to better reflect the activity within the tissue. In addition, the use of a flexible circuit board as the substrate meant that the entire device was flexible (Fig. 2i). Even when bent, each needle remained vertically aligned, demonstrating good mechanical toughness and stability. This design allowed the device to conform to the shape of the skin, improving the conformability of the electrode to the skin and reducing motion artifacts due to relative movement between the electrode and the skin, thus improving the quality of the recorded electrophysiological signals. The structure and morphology of the MMA were characterized using ultradeep three-dimensional (3D) microscopy (Fig. 2j). The microneedle tips were sharp, with a tip radius of approximately 10 μm, and the microneedle height was approximately 800 μm. To demonstrate the ability of the microneedles to penetrate the skin, a pig skin penetration test was performed. The microneedle tips were first stained with red fluorescent rhodamine B. The MMA was then applied vertically to the surface of the fresh pigskin, and a positive force was applied from top to bottom. After a period of 3 min, the microneedle array was removed, revealing red spots on the skin surface that resembled the arrangement of the MMA, with 3 mm between these red spots (Fig. 2k). When the skin was examined under a fluorescence microscope, an array of fluorescent spots was observed on the skin surface (Fig. 2l), indicating that the microneedles had indeed created micropores in the skin. In an ideal scenario, uniform force application would allow synchronous penetration of the skin tissue through all 32 channels. However, surface features of the skin, such as curvature and wrinkles, could result in uneven force distribution, potentially affecting the synchronous penetration of the 32 channels. For example, in areas where joints bend, it may be difficult to achieve uniform force application, resulting in partial or complete failure of certain channels to penetrate the tissue. Microscopic examination via SEM (Fig. 2m) clearly revealed micropores in the pigskin, with the microneedles creating circular holes 200 μm in diameter on the skin surface after penetration.

The penetration of these microneedles into the skin tissue was further investigated (Fig. 2n). After transdermal penetration, the pig skin was sliced into approximately 1 mm thick sections along the cross sections of the holes created by the microneedles after transdermal penetration. Fluorescence microscopy was used to image the areas penetrated by the MMA. It was observed that the diameter of the fluorescent areas corresponding to these needle punctures was approximately 200 μm, which closely matched the diameter of the microneedles. In addition, the penetration depth of the microneedle punctures was approximately 500 μm, which was shorter than the length of the microneedle tips (~800 μm). This could be due to the elasticity of the skin, which prevented the microneedles from fully adapting to the skin and left the basal parts of the needles partially exposed outside the skin tissue.

**Fig. 3:**
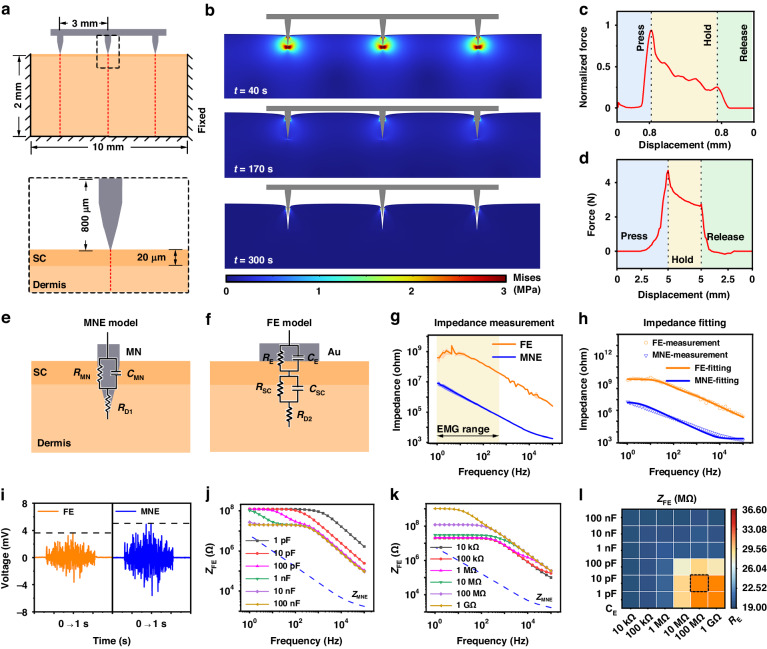
Mechanical and electrical simulation analysis of electrode–skin models. **a** Finite element models of microneedle penetration-retraction in skin. **b** Thermogram of the stress distribution in the skin during insertion and removal of the microneedle. **c** A plot of the reaction force versus loading displacement for the insertion and removal of microneedles was obtained through COMSOL simulations. **d** A plot of reaction forces versus loaded displacements was obtained by inserting and removing the MMA during actual mechanical testing. **e** Schematic diagram of the MNE-skin equivalent circuit model. **f** Schematic diagram of the FE-skin equivalent circuit model. **g** FE/MNE-skin impedance measurements. **h** Impedance fitting curves for MNEs versus FEs on the skin. **i** Electrical simulations were performed using typical EMG signals to obtain the EMG signals acquired by the MNE and FE models. The blue line represents the EMG signal detected by the MNE, while the orange line represents the EMG signal detected by the FE. **j** The ${Z}_{{FE}}$ of the FE model was obtained by varying the value of ${C}_{E}$ while ${R}_{E}$ was set at 100 MΩ. **k** The ${Z}_{{FE}}$ of the FE model was obtained by varying the value of ${R}_{E}$ while ${C}_{E}$ was set at 10 pF. **l** The heatmap summarizing the ${Z}_{{FE}}$ calculations conducted at a frequency of 100 Hz across varying ${R}_{E}$ and ${C}_{E}$ values

To evaluate the mechanical performance of the MMA, simulations of microneedle insertion and removal from the skin were performed using COMSOL Multiphysics. Based on the morphological characterization of the MMA, models for microneedle insertion and removal from the skin were established (Fig. 3a), with the skin partitioned into the SC (20 μm) and the dermis (2 mm)^33^. Due to the geometric similarity of the microneedle tips in the array, three microneedles were selected as representative for investigation. The microneedles were positioned 3 mm apart and had a height of 800 μm at the microneedle tips, with a base thickness of 200 μm. The microneedles were designated rigid bodies due to their considerably harder composition in comparison to skin. The skin was set as an elastic material and was simulated using the linear viscoelastic Kelvin–Voigt model with the specific parameters listed in Table S1^34^. Refining the skin edge mesh in the contact area captured subtle deformations, which allowed for precise simulation of skin deformations under large loads, avoiding deviations in size calculations. Fixed constraints simulated skin support by applying it to the side and bottom edges of the skin model. The contact between the microneedles and the skin followed a predetermined crack path, with a friction coefficient of 0.42 applied on the contact surfaces between the microneedles, the SC and the dermis^35^. Before applying the load, the microneedles were fixed in their initial positions. The microneedle array displacement was controlled to simulate the insertion-retraction process. The microneedles were vertically displaced along the −y-axis direction for the first 80 s, as per the reported microneedle insertion speed in the published literature^36^, by inserting the microneedles to a depth of 800 μm. The displacement was halted and maintained for 180 s before a vertical displacement was executed in the +y-axis direction, causing the microneedles to retract back to their original positions within 80 s. During this process, COMSOL Solid Mechanics was utilized to obtain the von Mises stress heatmap of the skin. (Fig. 3b). Notably, substantial deformation occurred in the region where the microneedle tips came into contact with the skin during insertion. The stress below the needle tips was concentrated, reaching 3 MPa, whereas on either side of the tips, it was approximately 1.5 MPa. As the skin underwent strain, cracks formed in the direction of the strain. During the static phase, the skin’s stress decreases over time due to the gradual restoration of cohesive forces in the skin tissue, leading to skin relaxation. During the removal phase, the stress gradually decreased until it approached 0 MPa, creating indentations in the skin where the microneedles had been placed. Moreover, the reaction forces that acted on the skin in the y-axis direction were acquired and then standardized (Fig. 3c). The insertion of microneedles led to a gradual increase in the reaction force due to the friction between the microneedles and the skin. At a depth of 600 μm, the reaction force rapidly increased and eventually reached its maximum at a depth of 800 μm. Once the microneedles ceased their motion and became lodged in the skin, relaxation ensued, resulting in a gradual reduction in the reaction force. Subsequently, as the microneedles were removed and the recovery process took place, the area of contact between the microneedles and skin decreased, leading to a gradual reduction in the reaction force, which eventually approached zero.

Additionally, pig skin was used as a substitute for human skin during mechanical tests to verify the precision of the simulation model and to obtain a better understanding of the mechanical interaction between the microneedles and the skin. The MMA device was attached to a force sensor probe during the tests, with an electric displacement stage used to regulate the vertical puncture of the MMA into the pig skin (Figure S3). The results depicted in Fig. 3d demonstrated a gradual increase in the counterforce during the insertion phase. The curve exhibited fluctuations at a counterforce of 1.5 N, which is indicative of the microneedle array piercing the pig skin. Afterward, the counterforce displayed a linear increase with an upward slope of approximately 1.8 N/mm after piercing the pig skin, reaching its peak at a depth of 5 mm, registering 4.7 N. Throughout the following static phase, the counterforce gradually decreased from 4.7 N to 3 N, indicating a modification in the adhesion classification between the microneedles and the skin. The counterforce was reduced until the microneedle array became detached from the skin, at 0 N.

When measuring electrophysiological signals, the contact impedance between the electrode and the interface affects the quality and stability of the signal conduction^37^. To investigate the electrical model of microneedle detection, an equivalent circuit model of the MNE-skin interface was constructed and compared with an FE model. In both cases, the skin components were represented using the traditional Cole model^38^. The equivalent interface models of the MNE-skin and FE-skin are shown in Fig. 3e, f^39–41^. The microneedle array penetrated the subject’s skin directly from the SC to the viable epidermis and was in direct contact with the interstitial fluid of the skin. This arrangement overcame the influence of the SC so that only the impedance of the epidermis was measured. ${R}_{{MN}}$ and ${C}_{{MN}}$ corresponds to the resistance and capacitance of the microneedle between the electrode and the viable epidermis. ${R}_{D1}$ represents the series resistance of the deeper tissue layers. The equivalent impedance of the MNE-skin interface is given by Eq. 1.

1 $${Z}_{{MNE}}=\frac{{R}_{{MN}}}{\sqrt{1+{\left(\omega {R}_{{MN}}{C}_{{MN}}\right)}^{2}}}+{R}_{D1}$$

The circuit model for the FE-skin was much more complex than the circuit model for the MNE-skin. The combination of ${R}_{E}$ and ${C}_{E}$ corresponded to the interface impedance between the electrode and the skin, while the combination of ${R}_{{SC}}$ and ${C}_{{SC}}$ corresponded to the impedance characteristics of the SC^42^. ${R}_{D2}$ represents the series resistance of the deeper tissue layers. The equivalent impedance of the FE-skin interface is given by Eq. 2.

2 $${Z}_{{FE}}=\frac{{R}_{E}}{\sqrt{1+{\left(\omega {R}_{E}{C}_{E}\right)}^{2}}}+\frac{{R}_{{SC}}}{\sqrt{1+{\left(\omega {R}_{{SC}}{C}_{{SC}}\right)}^{2}}}+{R}_{D2}$$

To compare the skin-electrode impedance between the MNE and FE, a custom-made FE with a diameter of 0.5 mm and the same contact area as the MNE was fabricated. The skin-electrode impedance of both the MNE and the FE was tested separately. These electrodes were placed on the surface of the skin on the back of New Zealand rabbits with a distance of 3 cm between them. An electrochemical workstation was used to measure the skin-electrode impedance for both electrodes. Three sets of data were obtained, and the average impedance and standard deviation were calculated (Fig. 3g). The impedance magnitude decreased with increasing frequency, with the impedance of the MNE being two orders of magnitude less than that of the FE. At 1 kHz, the average impedance of the MNE was 29.87 ± 3.24 kΩ, while that of the FE was 18.31 ± 2.45 MΩ. To further explore the skin-electrode model quantitatively, equivalent circuits were used for plotting. Actual measurement data from MNE-skin and FE-skin interfaces were imported, and corresponding equivalent circuit models were created. Parameter fitting was employed for the imported impedance data, with the goal of achieving the closest possible match between the calculated impedance from the model and the actual measured impedance (Fig. 3h). The necessary parameters for the circuit model were obtained via this process.

To compare the differences in electrophysiological signals detected by the FE and MNE more clearly, a circuit was built to simulate the electrode–skin interface using Multisim software. The specific parameters achieved through the fitting process were integrated into the circuit (Fig. S5-1b). Typical EMG signals were used for testing, and the simulated EMG signals detected by the FE and MNE were obtained (Fig. 3i). The detected signal from the MNE revealed a greater amplitude than that from the FE, with the MNE detecting a maximum signal value of 5 mV and the FE detecting a maximum signal value of 3.6 mV. The root mean square (RMS) amplitude reflects the amplitude variations in surface EMG data. The MNE had a maximum RMS value of 1.8 mV, while the FE had a maximum RMS value of 1.2 mV (Fig. S5-1c). The SC of the skin has sebaceous glands that excrete sebum, which may affect the detection of electrophysiological signals using FEs^43^. As a result, it is necessary to consider the interference of skin surface secretions on electrodes while measuring electrophysiological signals with FEs, as they can impact the contact between the electrode and the skin surface. The skin’s connection to the electrode altered the values of resistance and capacitance at the contact point, impacting the total impedance of the FEs. To investigate the impact of differing levels of connection on the total impedance of FEs, simulations with various contact states altered ${R}_{E}$ and ${C}_{E}$ values. Multisim software was used to test multiple values of ${R}_{E}$ and ${C}_{E}$ to simulate a range of contact situations. The researchers calculated the total impedance ${Z}_{{FE}}$ of the FE-skin interface. They observed that while keeping ${R}_{E}$ constant, an increase in ${C}_{E}$ (Fig. 3j) resulted in a decrease in the impedance ${Z}_{{FE}}$. The impedance variation exhibited a gradual trend ranging from 0 to 100 Hz and manifested a linear reduction with an approximate slope of −1031.85 Ω/Hz from 100 Hz to 100 kHz. A reliable value demonstrated that an increase in ${R}_{E}$ led to a proportional increase in impedance. This tendency progressed steadily from 0 to 500 Hz and then displayed a linear decrease with an estimated gradient of −347.96 Ω/Hz from 500 Hz to 100 kHz (Fig. 3k). ${Z}_{{FE}}$ was affected by the attachment state of the FEs, as evidenced in Fig. 3l and Fig. S5-2. Changes in ${C}_{E}$ had a negligible impact on ${Z}_{{FE}}$ when ${R}_{E}$ was less than 1 MΩ. Similarly, alterations in ${R}_{E}$ had an insignificant effect on ${Z}_{{FE}}$ when ${C}_{E}$ exceeded 10 nF. However, when ${R}_{E}$ exceeded 1 MΩ, ${Z}_{{FE}}$ was noticeably affected by ${C}_{E}$ variations between 1 pF and 100 pF. Insufficient adherence between the skin and FEs resulted in a total increase in impedance, which could reduce the quality of electrophysiological signal detection. On the other hand, the MNE can penetrate the skin, diminishing the effect of skin surface conditions on total impedance. Although high-input impedance amplifiers were utilized, the electrode–skin contacts still influenced the signal quality to a certain extent. Even though the amplitude error may not be significant, variations in the contact impedance can still affect the signal’s frequency response and noise level. Hence, MNEs offer more dependable signal detection capabilities and improved signal quality compared to FEs.

**Fig. 4:**
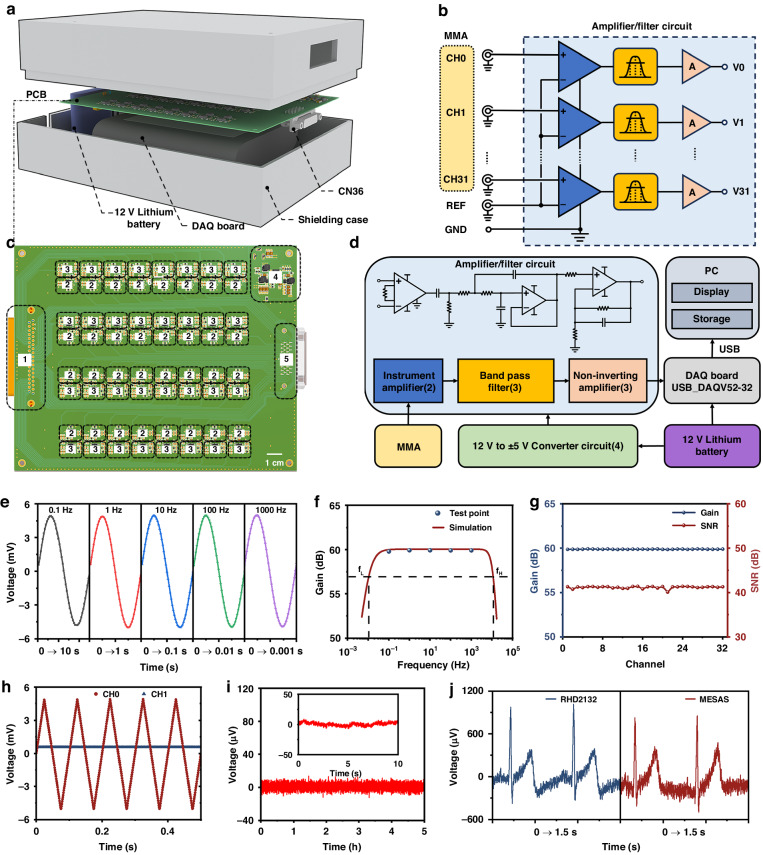
Design and validation of MESAS. **a** Exploded view of the MESAS, consisting of a shielding case, printed circuit board (PCB), 12 V lithium battery and DAQ board. **b** Wiring diagram of an amplifier/filter circuit was provided, in which thirty-two working electrodes shared a common reference electrode. **c** PCB layout design (top). The dashed boxes indicate the locations of the modules. **d** System block diagram of the MESAS. **e** Test signals of different frequencies were applied to one channel, and the amplified and filtered signals were detected. The gain was measured and compared with the frequency response simulation plot. **f** The frequency response of the amplifier/filter circuit was simulated with upper and lower frequencies set at 10 kHz and 0.01 Hz, respectively. **g** For the same test signal, the gain and SNR of 32 channels were measured. **h** A ± 5.0 mV, 100 Hz triangular wave was applied to one channel, and the signals acquired in that channel and the neighboring channels were detected. No crosstalk was observed in the neighboring channels. **i** Five hours of continuous basal noise detection. Inset: 10 s of basal noise. **j** Raw ECG data from Ag/AgCl gel electrodes were acquired using the MESAS and a commercial instrument

A MESAS (Fig. 4a and Fig. S6-3) was developed to efficiently capture electrophysiological signals in conjunction with the MMA device. As human electrophysiological signals are typically weak, signals acquired from electrodes are often influenced by noise during signal transmission. To convert electrophysiological signals into high-quality data, amplification and filtering of the signals is needed, which is achieved through the use of an amplification and filtering circuit (Fig. 4b). The amplifier/filter circuit consisted of instrumentation amplifiers, bandpass filters and noninverting amplifiers. To minimize attenuation of the input signals, a high-input impedance AD620 instrumentation amplifier was used for initial amplification (gain of 10). An RC high-pass filter (cutoff frequency of 0.01 Hz) was used to remove any baseline drift that might have occurred during detection. Since most of the useful electrophysiological signal information is concentrated in low-frequency components, it was necessary to enhance the circuit’s ability to reject high-frequency noise. A Butterworth low-pass filter (cutoff frequency of 10 kHz) was used to effectively suppress high-frequency noise. The signal was then further amplified by a noninverting amplifier (gain of 100) and acquired by the analog-to-digital converter (ADC) of a data acquisition card with a voltage acquisition range of ±5 V. The operational amplifiers used in both the bandpass filter circuit and the postamplification circuit are OPA2209, known for their unity-gain stability and ultralow voltage noise, making them highly suitable for voltage amplification circuits. To reduce wiring complexity, minimize cabling and simplify the connection of the electrophysiological measurement system, the IN+ terminals of the 32-channel instrumentation amplifiers were connected to the MMA unit to receive electrophysiological signals from all 32 channels. The IN- terminals of the instrumentation amplifiers were connected together to act as a reference electrode (REF) for the electrophysiological signals, which were then connected to a reference point on the body. The REF pins of the instrumentation amplifiers were also connected to the ground electrode (GND), ensuring the correct potential difference between the input and output signals of the amplifier relative to the ground and helping to suppress power noise and interference.

The system diagram is illustrated in Fig. 4d, and the specifications are listed in Table S2. A 12 V rechargeable lithium-ion battery was used as the system power input to mitigate interference from power-frequency signals and to ensure a stable power supply to the data acquisition (DAQ) board. A two-channel DC/DC converter LT8471 was used for voltage conversion to provide ±5 V for the amplification and filtering circuitry. The DAQ board performed real-time sampling of the amplified multichannel signals at a frequency of 10 kHz and was connected to an external computer via a USB cable. Customized LabVIEW software was used for waveform display and data storage (Fig. S6-4). The data were stored in the TDMS file format to facilitate subsequent signal processing within the LabVIEW program. In addition, to minimize interference from external noise sources, the acquisition system was enclosed in a shielding case, and a shielded CN36 cable was used to connect the sensor. This shielding helped to maintain the integrity of the acquired signals and reduce external interference.

Following the construction of the multichannel electrophysiology acquisition system, performance testing was carried out to verify its compliance with design expectations. As shown in Fig. 4e, a sine wave with an amplitude of 10 mV was introduced to the 32-channel input to evaluate the amplifier/filter circuit gain at various frequencies. The resulting output signals are depicted in Fig. 4f. The gain remained stable between 0.1 Hz and 1 kHz, consistent with the previously obtained frequency response through simulation software (Fig. 4g). The voltage gain and signal-to-noise ratio (SNR) of the 32-channel amplification and filtering circuitry were tested with a 10 Hz sine wave frequency. As demonstrated in Fig. 4g, all channels exhibited a gain of 60 dB and good consistency across channels. Furthermore, all channel SNRs exceeded 40 dB, satisfying the criteria for detection. Subsequently, a ±5.0 mV, 100 Hz triangular wave was input to channel 1, and adjacent channel interferences were assessed. Figure 4h presents evidence of minimal interference between neighboring channels. Following individual testing of each channel, no abnormal or interfering signals were observed, indicating a lack of crosstalk among the multiple channels. The stability of the instrument during long-term measurements was confirmed by shorting the working electrode and reference electrode and continuously monitoring recorded electrical signals for a duration of 5 h (Fig. 4i). To evaluate the dependability of the MESAS, a comparison was made with a commercial device. The same parameters, including the sampling rate and filtering frequency, were applied to both systems. Following this, they were simultaneously connected to a single test subject utilizing the single-lead connection method, with commercially available Ag/AgCl electrodes used for electrocardiographic detection. As illustrated in Fig. 4j, both the MESAS and the commercial device detected electrocardiographic signals effectively, with comparable amplitudes. Nevertheless, the MESAS demonstrated increased levels of noise. Hence, these outcomes show that the MESAS was proficient in precisely detecting electrophysiological signals.

**Fig. 5:**
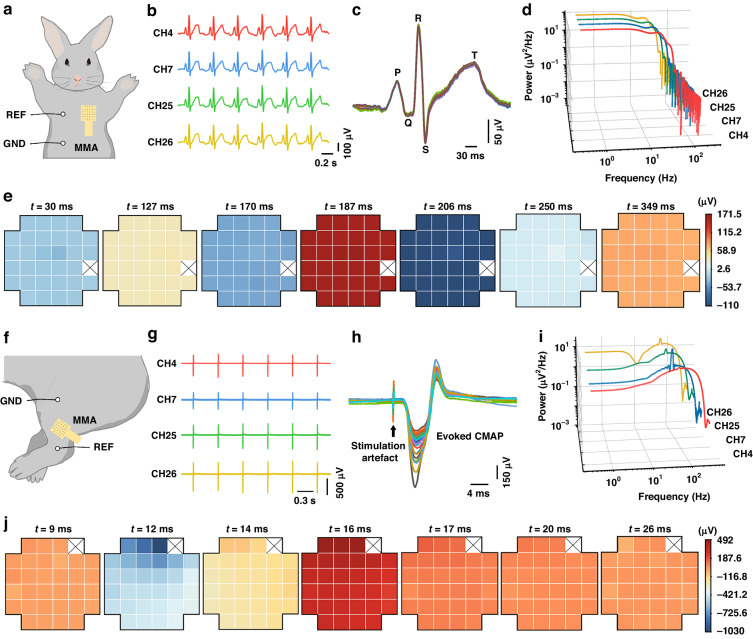
ECG and EMG signals were detected in New Zealand rabbits using the MMA. **a** Position of the MMA electrode on a rabbit model when used to record the ECG signal in differential acquisition mode. **b** ECG signal fragments from 4 channels. **c** Overlay of ECG signals detected by 31 channels. **d** PSD plot of the recorded ECG signals. **e** The changes in the amplitude of the ECG signal at each stage were analyzed. (× indicates that no electrophysiological signal was detected.) **f** Position of the MMA electrode on a rabbit model when used to record the EMG signal in differential acquisition mode. **g** EMG signal fragments from 4 channels. **h** Overlay of EMG signals detected by 31 channels. **i** PSD plot of the recorded EMG signals. **j** Changes in the amplitude of the EMG signal at each stage were analyzed (× indicates that no electrophysiological signal was detected)

Animal experiments were carried out on healthy New Zealand rabbits. The rabbits were anesthetized, and their chest hair was removed. First, ECGs were measured using the MMA, with reference electrodes placed in the upper right chest and the ground electrode on the lower right abdomen, as depicted in Fig. 5a. Except for one MNE that was damaged, every MNE in the MNA successfully recorded the ECG signals. Figure 5c shows the overlaid signs of a single ECG from 31 channels, demonstrating that the identified ECG signals had reliable patterns with fairly high magnitudes and distinct ECG characteristic peaks (P, Q, R, S, T). The power values of the ECG signals within each frequency range were calculated based on the signals recorded by MNEs. A power spectral density (PSD) plot (Fig. 5d) was generated to illustrate the energy distribution of the ECG signals across different frequency ranges. The PSD plot of the ECG signals demonstrated greater power in the lower-frequency range (0.5–50 Hz). Each ECG signal was sectioned into seven phases, and the magnitudes of every phase were assessed for statistical significance. A heatmap was plotted to illustrate the spatial distribution of ECG signal amplitudes over multichannel MNEs based on each phase (Fig. 5e). The results revealed that the signals recorded on the 31 electrodes were mostly synchronous, primarily due to the consistent rhythm of the heartbeats. Slight variations in recorded ECG signal amplitudes could be traced back to differences in the attachment of the needle electrodes to the surface tissue. Moreover, continuous monitoring of ECG signals for 45 min was conducted using the MMA (Fig. S9-2). The results demonstrated that the MMA could continuously and stably monitor the signals, and the signals from multiple channels exhibited excellent synchronicity.

The next step was to validate the MMA for transdermal detection of multichannel EMG signals. Specifically, the MMA was inserted into the quadriceps femoris muscle of the thigh to detect EMG signals in this muscle region. EMG signals from the quadriceps femoris muscle can provide physiological information about muscle activity levels, motor control and fatigue states. When rabbits were placed under general anesthesia, their leg muscles became unresponsive, which prevented the direct detection of EMG signals. Therefore, to record the EMG signals generated in the leg muscles, the sciatic nerve received appropriate electrical stimulation to induce muscle contractions in the rabbits’ legs. The MMA was fixed onto the pertinent leg muscles to compute the subcutaneous compound muscle action potentials (CMAPs). Reference electrodes were positioned at the knee joint. With the implementation of electrical pulse stimulation (at a current amplitude of 2 mA, a frequency of 2 Hz, and a pulse width of 100 µs), consistent contractions of the leg muscles were observed, and a range of EMG signals were recorded by the MMA (Fig. 5g). Figure 5h shows the combined EMG signals taken from 31 channels, depicting sharp peaks as stimulation artifacts followed by evoked CMAP signals. The PSD plot of the EMG signals revealed more energy in the lower-frequency range, usually 20–250 Hz (Fig. 5i). The individual EMG signals were divided into seven phases, and the amplitudes of each phase were subjected to statistical analysis. A heatmap was plotted to illustrate the spatial distribution of EMG signal amplitudes across the electrodes with multiple channels, incorporating the amplitudes from each phase (Fig. 5j). There were minor disparities in EMG signal amplitudes observed in various channels, which could be due to the variation in MNE placement in regions of active muscle movement. These results indicated that the MMA possessed the potential for the detection of EMG signals.

**Fig. 6:**
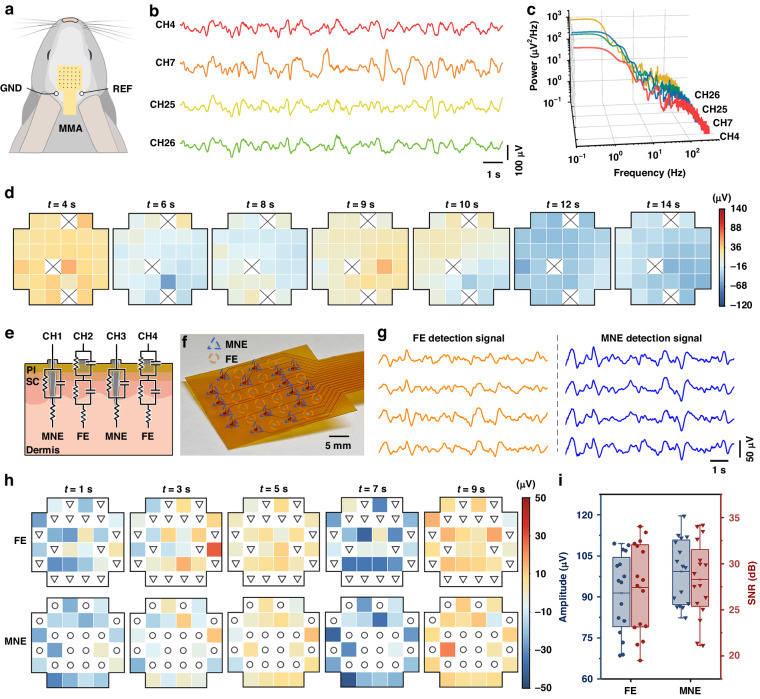
EEG signals were detected in New Zealand rabbits using the MMA. **a** Position of the MMA electrode on a rabbit model when used to record EEG signals in differential acquisition mode. **b** EEG signal fragments from 4 channels. **c** PSD plot of the recorded EEG signals. **d** The changes in the amplitude of the EEG signal at each stage were analyzed. (× indicates that no electrophysiological signal was detected.) **e** Schematic diagram of the electrode-skin equivalent circuit model, which includes MNEs and FEs. **f** Optical image of a device with integrated FEs and MNEs. The blue triangles indicate the corresponding positions of MNEs, and the orange circles indicate the corresponding positions of FEs. **g** Segments of EEG signals were recorded separately for FEs and MNEs. **h** The EEG signals recorded from the MNEs and FEs were separately analyzed for changes in amplitude at each stage. **i** Comparison of the amplitudes and SNRs recorded by MNEs and FEs

The next step was to validate the MMA for transdermal detection of multichannel EEG signals. The MMA was inserted into the cranial region of the rabbit to detect EEG signals below the scalp. EEG signals beneath the cranial scalp can be used to analyze electrical activity in the brain, diagnose neurological disorders and reflect cognitive and emotional states. The EEG signals were detected using the MMA device (Fig. 6a), and Fig. 6b presents representative signals spanning several seconds. The EEG signals were transformed into power spectra to observe the distribution of signals at different frequencies. From the PSD results (Fig. 6c), it can be observed that the EEG signals recorded by MMA exhibited a greater distribution in the low-frequency range. Because the New Zealand rabbits were under anesthesia, the recorded EEG signals tended to display slow waves, possibly due to the inhibitory effect of anesthesia on the nervous system^44^. The signals were divided into seven phases, and the amplitudes of each phase were analyzed statistically. A heatmap was plotted to illustrate the spatial distribution of EEG signal amplitudes across the multichannel electrodes using the amplitudes of each phase (Fig. 6d). The results indicate that the recorded EEG signals had different voltage values in different areas where the MMA was attached, indicating spatial distribution differences of the EEG signals under the areas covered by the MMA. EEG signals were not detected in three MNEs, possibly due to insufficient penetration of the epidermis or poor contact between the electrodes and the epidermal layer. To compare the simultaneous recording of EEG signals from the MNE and FE under identical experimental conditions, a schematic diagram of a device containing both types of electrodes with alternating layouts was created (Fig. 6e) for simultaneous EEG signal detection. The MMA was designed with 16 MNEs and 16 FEs. These electrodes were arranged alternately, with a distance of 3 mm between adjacent electrodes. The fabricated MNE-FE electrode array device is shown in Fig. 6f. The results of the simultaneous recording process using MNEs and FEs are displayed in Fig. 6g. The electrical signals captured by the MNEs closely resemble those obtained by the FEs, albeit with marginally greater amplitudes in the MNE recordings. This phenomenon can be attributed to the inherent capacity of MNEs to puncture the SC and establish improved electrical communication with subcutaneous tissues, thereby ensuring superior signal transmission. The EEG signals were partitioned into five phases, and the amplitudes of each phase were subjected to statistical analysis. A heatmap was plotted to demonstrate the spatial distribution of EEG signal amplitudes on the MNE and FE (Fig. 6h). The top row shows the spatial distribution of EEG recorded by the FE, with 16 electrodes successfully capturing EEG signals. The lower row shows the spatial distribution of EEG recorded by the MME, with 16 MNEs successfully capturing the EEG signals. The comparison of the signal amplitudes and SNRs recorded by the FEs and MNEs (Fig. 6i) reveal that the MNEs recorded greater EEG signal amplitudes and SNRs than did the FEs. The average amplitude of the EEG signal measured by the MNEs was 99.27 ± 11.61 μV, while the average amplitude of the EEG signal recorded by the FEs was 91.47 ± 14.10 μV. The MMEs recorded EEG signals with an average amplitude that is ~7.80 μV greater than that recorded by the FEs. Notably, there was considerable variance in signal amplitudes among the FEs, which may be attributed to variations in their attachment to the skin. The average SNR for the EEG signals measured by the MNEs was 27.42 ± 4.70 dB, while the average SNR for the EEG signals recorded by the FEs was 28.27 ± 4.07 dB. This experiment showed that MNEs performed better than FEs when recording multichannel EEG signals, resulting in higher signal amplitudes and SNRs under identical conditions.

## Conclusions

In this study, a microneedle array device comprising 32 independent channels on a single patch was demonstrated. This device was fabricated on a flexible circuit board utilizing individual electrode functionalization and assembly techniques. Each microneedle possessed electrophysiological sensing capabilities, enabling the simultaneous collection of electrophysiological signals from multiple locations and thus achieving high-throughput data acquisition. The MMA conforms seamlessly to the curved human skin surface because of its flexible substrate. Additionally, the microneedles can penetrate the SC for direct contact with the interstitial space, which reduced the skin-electrode impedance and improved the signal acquisition efficiency. A circuit system that allows multichannel and high-frequency potentiometric signal acquisition was developed to support the operation of the MMA, which collects, stores, and displays the recording results of electrophysiological signals. The experimental results demonstrated that the microneedle array device can successfully record ECG, EMG, and EEG signals in a rabbit model and can effectively distinguish the spatial distribution profile of these electrophysiological signals. Moreover, the EEG signals recorded by MNEs presented better signal quality than did the signals recorded by conventional flat dry electrode arrays, suggesting that the MMA enables transcutaneous recording by lowering the skin-electrode impedance. This work provides a promising strategy for improving the signal quality of dry electrodes for transdermal electrophysiological signal acquisition in a high-throughput manner. The development of this technology can provide useful tools for the field of human‒machine interface technology and may benefit the development of recording tools for electrophysiology-related diseases.

## Materials and methods

### Fabrication and characterization of MMA

Stainless steel needles (type 201, 200 μm diameter) were cut by laser machining (Han’s Laser Technology Industry Group Co., Ltd.), leaving only the tip portion of the needle (approximately 1 cm in length). Acidic detergent was utilized to effectively remove the oxide layer from the needle surface. The treated stainless steel needles were immersed in a sodium gold sulfite solution (Yuncaitaotao Company) and plated using an electrochemical workstation (CH Instruments, Inc.) at a current of −2 mA for 10 min, resulting in a thin gold layer on the surface of the needles.

After the flexible circuits were designed, printed circuit processes, including photolithography, etching, drilling, plating and laser cutting, were used to prepare the flexible circuit boards by Shenzhen Jialichuang Technology Group Co., Ltd. First, the copper foil substrate underwent precision cutting to achieve the desired pattern. Subsequently, specific locations were selected for hole drilling and plating, resulting in the creation of the intended via holes. Next, a photoresist was applied onto the copper foil substrate and subjected to precise exposure. The subsequent development process, involving immersion in a solution, led to the formation of patterns encompassing pad arrays, leads, and other essential features. The removal of excess copper foil was carried out via chemical etching in a dedicated solution, and the dry film was then peeled away to reveal the meticulously crafted circuit pattern. To enhance resistance against corrosion and oxidation, chemical plating of gold was employed on the pads, while the leads were insulated using PI insulating material.

To integrate the microneedles with the flexible circuit board, an 800 μm thick FR4 substrate containing thirty-two vias was prepared using a PCB fabrication process (Shenzhen Jialichuang Technology Group Co., Ltd). The flexible circuit board was positioned atop this FR4 substrate, and thirty-two microneedles were inserted vertically into the corresponding microholes within the FR4 substrate. Connections between the microneedles and the pads of the flexible circuit board were established using stainless steel solder (Dongguan Yarun Hardware & Electronics Co., Ltd). Excess portions of the microneedles were removed using diagonal pliers, and the remaining microneedles were individually welded to the flexible circuit board. Subsequently, the flexible circuit board was separated from the FR4 substrate, revealing an integrated array of microneedles, each approximately 0.8 mm in length.

### Surface morphology characterization of the electrodes

The super depth of a field microscope (VHX-7000, Keyence, Japan) and SEM (SUPRA 60, Zeiss) were utilized to observe the surface morphology and microstructure of the MMA. A cotton swab was used to evenly apply a 2 mg/mL solution of Rhodamine B in water onto the microneedle tips. Subsequently, the microneedle tips were placed vertically on the surface of fresh pig skin and inserted with force. After a 3-min incubation period, the microneedles were removed, and the skin was examined using a fluorescence microscope (MF41, MSHOT) to assess the transdermal treatment by the microneedles. To validate the transdermal performance of the microneedle tips, cross sections of pig skin were prepared with a thickness of approximately 1 mm along the holes created by the microneedles. Imaging of the regions penetrated by the MMA was conducted using fluorescence microscopy. To examine the pores in the skin post-transdermal delivery, pig skin squares containing microneedle puncture sites approximately 1 cm^2^ in size were excised with a surgical blade. Subsequently, they underwent dehydration, drying, and gold sputter coating and were finally observed using SEM to assess the penetration of the pig skin.

### Mechanical simulation via COMSOL multiphysics

The simulation model was built using COMSOL Multiphysics 6.1, employing the solid mechanics module to construct the transdermal microneedle model. Based on the morphological characterization of the microneedle array sensor, a two-dimensional finite element model was developed for the insertion and removal of microneedles from the skin. Due to the high hardness and elastic modulus of the SC and the similarity in parameters between the subcorneal region and the dermis layer, the skin model was simplified to include a 20 μm thick SC and a 2 mm thick dermis layer. Considering the geometric symmetry of the microneedle array, a study was carried out using three microneedles with a needle-to-needle distance of 3 mm. The shape of the needle tip was simplified to a trapezoid with a 200 μm base and a 2 μm tip radius, and the height of the needle tip was 800 μm. To obtain the mechanical distribution within the transdermal microneedle model, a transient analysis of the solid mechanics physics field was added. The transient equation of solid mechanics describes the response and deformation of solid objects over time. It is based on the principles of conservation of mass and Newton’s second law, combined with the theory of elasticity, to study the dynamic behavior of objects under external forces. The transient equation of solid mechanics can be written as follows:

$$\rho \frac{{\partial }^{2}u}{{\partial t}^{2}}=\nabla \cdot {\left({FS}\right)}^{T}+{F}_{v}$$

where ρ (SI unit: kg m^−3^) is the density of the object, t (SI unit: s) represents time, $u$ (SI unit: m) denotes the displacement vector, *F* represents the deformation gradient tensor, ${F}_{v}$ (SI unit: N) represents the externally applied volume force, and S (SI unit: Pa) represents the stress tensor.

In the solid mechanics node, attributes and conditions related to the transient analysis have been set up. Due to the significantly greater stiffness of the microneedle compared to that of the skin, the microneedles were modeled as rigid bodies. The skin was defined as an elastic material, and a linear viscoelastic Kelvin–Voigt model was used for the simulation. Fixed constraints were applied to the side and bottom edges of the skin model to simulate the support provided by the skin. The contact boundary between the skin and the microneedle was defined as a contact pair, and three types of contact behaviors, namely, separation, adhesion, and friction, were added to accurately simulate the interaction between the skin and the microneedle. The adhesive Poisson’s ratio and the friction coefficient of the contact pair were set to 0.48 and 0.42, respectively. Loading conditions were applied to the microneedle, causing it to insert into the skin at a speed of 10 μm/s in the vertical direction. After reaching a depth of 800 μm, it remained stationary for 180 s and then was removed from the skin at a speed of 10 μm/s. The mesh in the skin contact area was refined to ensure sufficient mesh refinement in the microneedle-skin contact area to accurately simulate the deformation of the skin under large loads and to avoid size-related distortions. The contact between the microneedle and the skin followed a predetermined crack path. The solver was used to obtain the von Mises stress thermal map of the skin and the reverse forces in the y-axis direction.

### Mechanical testing of the MMA

A force gauge (HP-20, Yueqing Handpi Instruments Co., Ltd) was attached to a motorized displacement table (GCD-203050M, Daheng). The force gauge was securely affixed to the displacement table, and the force gauge probe was precisely aligned in a vertical orientation with respect to the working area below. A double-sided adhesive was used to secure the MMA onto the dynamometer probe, ensuring that the adhesive firmly bonded the MMA to the probe and that the microneedle was vertically aligned with the table.

A piece of fresh pig skin was placed directly beneath the dynamometer, and the motorized displacement stage was controlled so that the MMA penetrated the skin in a downward vertical direction at a rate of 0.29 mm/s until the MMA successfully pierced the skin. Once penetration was achieved, a pause of 3 s followed, after which the MMA was controlled to rise vertically upward at the same speed as the piercing process until it was completely detached from the skin. Throughout the experiment, the dynamometer recorded the overall stress conditions of the MMA, including the variations in force values during both the puncturing and detaching processes.

### FE-skin and MNE-skin impedance measurements

Electrochemical impedance spectroscopy (EIS) was conducted using an electrochemical workstation (CH Instruments, Inc.) within the frequency range of 1 Hz to 100 kHz. Two microneedle tips of MNEs were positioned on the backs of New Zealand rabbits with a spacing of 3 cm, and the depth of insertion of the microneedle tips into the epidermis was controlled to be ~800 μm. Before applying the electrodes to the skin, the skin surface was cleaned with 75% medical alcohol. Subsequently, the same procedure was performed with FEs for testing. Three measurements were taken for each type of electrode, and the results were averaged to minimize the impact of instrument noise and electrode movement. The impedance of the wires and the impedance of the skin tissue between the two electrodes were disregarded in the testing circuit.

### Electrical simulation of the FE and MNE for EMG detection

For the FE-skin model, when the electrode was in contact with the skin, a metal-electrolyte interface between the electrode and sweat or oil was created. In this case, ${R}_{E}$ and ${C}_{E}$ were used to calculate the impedance associated with the electrode-skin interface. Given that the SC consisted of nonconductive dead cells that acted as insulators between the electrode and the living, conductive tissue within the skin, the impedance of the SC was described using a capacitor model denoted by ${R}_{{SC}}$ and ${C}_{{SC}}$. The series resistance of the internal tissue was represented as ${R}_{D2}$. In contrast, the MNE-skin model established a connection through the skin’s SC to the layer where hair growth occurred. It was in direct contact with the electrolyte within the skin, effectively bypassing the influence of the skin’s SC. In this case, only the impedance ${R}_{{MN}}$ and the contact capacitance$\,{C}_{{MN}}$ between the electrode and the hair growth layer were present, with ${R}_{D1}$ representing the series resistance of the internal tissue. The electrical signal transmission process was simulated using Multisim software (National Instrument Corp.). Two electrode–skin equivalent models were constructed and connected to an operational amplifier circuit. Typical EMG signals were introduced using a PWL voltage element. Using an AD620 operational amplifier, one electrode-skin equivalent model was connected to each of the positive and negative inputs, with the gain set to 1. An oscilloscope was utilized to record the waveforms detected by both models. Based on actual measurement data of the MNE-skin and FE-skin interfaces, the data were imported into ZView (Scribner Associates Inc.) fitting software for data fitting. The parameters of the two electrode–skin equivalent models were determined as follows: ${R}_{E}=7.246\times {10}^{8}{{\Omega}}$, ${C}_{E}=\,1.198\times {10}^{-11}{\rm{F}}$, ${R}_{{SC}}=\,1.729\times {10}^{7}{{\Omega}}$, ${C}_{{SC}}=\,1.672\times {10}^{-11}{\rm{F}}$, ${R}_{D2}=\,5.145\times {10}^{4}{{\Omega}}$, ${R}_{{MN}}=5.681\times {10}^{6}{{\Omega}}$, ${C}_{{MN}}=\,8.769\times {10}^{-9}{\rm{F}}$, and ${R}_{D1}=\,2.473\times {10}^{3}{{\Omega}}$. The RMS amplitude of the EMG signal was calculated using the Biosignal RMS VI in LabVIEW.

### FE-skin model impedance simulation

The parameters of the FE-skin model were simplified by setting ${R}_{{SC}}=\,2\times {10}^{7}{{\Omega}}$, ${C}_{{SC}}=\,2\times {10}^{-11}{\rm{F}}$, and ${R}_{D1}=\,5\times {10}^{4}{{\Omega}}$. The values of ${R}_{E}$ and ${C}_{E}$ at the contact interface were used to calculate the total impedance with different combinations of resistance and capacitance at the contact interface. The capacitive and resistive components were configured in Multisim and connected to the FE-skin model circuit. AC analysis was performed to calculate the total impedance of the FE-skin model. The frequency range was set from 1 Hz to 100 kHz.

### Design and development of the MESAS

The self-developed MMA system consisted of a PCB, a data acquisition card, a 12 V lithium battery, a shielding case, and a customized program. The PCB integrated 32-channel amplification and filtering circuits as well as power supply circuits. The amplifier/filter circuit included an instrumentation amplifier, a bandpass filtering circuit, and a noninverting amplifier. The instrumentation amplifier AD620 (Analog Devices) with a high common-mode rejection ratio was used to measure the difference between two electrophysiological signals and amplify them by a factor of 10. The signal was then passed through a bandpass filter consisting of an RC high-pass filter and a Butterworth low-pass filter. The high-pass filter circuit (cutoff frequency of 0.01 Hz) attenuated the DC component in the signal, and the low-pass filter circuit (cutoff frequency of 10 kHz) removed high-frequency noise. The noninverting circuit then amplified the filtered signal by a factor of 100. OPA2209 (Texas Instruments) operational amplifiers were used in both the bandpass filter circuit and the noninverting amplifier. The power supply circuit used a dual-channel DC/DC converter LT8471 (Linear Technology Corporation) for voltage conversion, providing a ±5 V power supply to the amplifier/filter circuit. The DAQ board (USB DAQ v5.2_32, HKTECH Co. Ltd.) was used to collect the output of the amplifier/filter circuit, enabling 32 channels to be completely synchronized with a maximum synchronous sampling rate of 50 kSPS. A 12 V lithium battery (Shenzhen Beiliang Battery Technology Co., Ltd) served as the system’s power input, reducing the influence of power frequency noise and providing a cleaner power supply to the amplifier/filter circuit. The shielded case was used to improve the noise immunity of the system. The shielding case was designed as a Faraday cage and consisted of a metal shield case measuring 241 mm in length, 170 mm in width, and 80 mm in height (Shenzhen Yuexinwang Plastic Hardware Machinery Products Co., Ltd.). A CN36 shielded cable (FT-SC100C, Fu Tai Technology) was used to connect the MMA sensor, effectively reducing the impact of electromagnetic interference. The customized program was developed based on LabVIEW and was primarily used to display and store the collected electrophysiological signals.

### Characterization of the supporting circuit for the MESAS

To verify the amplifier/filter circuit, a waveform generator DG 1032Z (RIGOL, China) was employed to generate both a sine wave and a triangle wave with an amplitude of 10 mV. These waveforms were connected to the input of the amplification filter circuit. A custom LabVIEW program for signal acquisition was used, and the Extract Single Tone Information VI was utilized to extract the signal component with the largest amplitude from the acquired signal. The amplitude ratio with respect to the input signal of the amplification filter circuit was calculated, providing the voltage gain of the circuit under test for different frequencies. Simultaneously, the amplifier/filter circuit was constructed via the Multisim program for frequency response simulation analysis. The simulation results were then compared with the actual test results to evaluate the performance of the amplifier and filter circuit. Additionally, the SINAD Analyzer VI was applied to obtain the SNR for all channels. To validate the accuracy of the MESAS system’s signal detection, both commercial electrophysiological acquisition systems (RHD 2132 amplifier board and RHD 2000 USB interface board, Intan Technologies) and the MESAS system were simultaneously employed to ECG signals from the same test subject.

### Signal processing and statistical analysis

Electrophysiological signal processing was performed by a custom LabVIEW program using the Advanced Signal Processing Toolkit for signal processing and analysis. For ECG recordings, WA Detrend VI was used to remove baseline drift, and Filter VI was applied to implement a bandpass filter from 1 to 150 Hz along with a smoothing filter. For EMG recordings, a bandpass filter ranging from 1 to 400 Hz and a smoothing filter were applied using Filter VI. In the case of EEG recordings, Filter VI was utilized to apply a bandpass filter from 0.5 to 50 Hz along with a smoothing filter. All PSDs were analyzed using the FFT power spectrum and PSD VI. All the statistical analyses were conducted and visualized using Excel and Origin 2023.

The SNR was calculated as follows:

$${SNR}({dB})=20\,\times {\mathrm{log}}10\frac{{{RMS}}_{{Signal}}}{{{RMS}}_{{\rm{Noise}}}}$$

Here, ${{RMS}}_{{Signal}}$ and ${{RMS}}_{{\rm{Noise}}}$ are the RMS values of the EEG signal and baseline noise, respectively. The RMS value was calculated using the basic average DC-RMS VI.

### Recording of electrophysiological signals in experimental animals

For EMG detection, an approximately 2 cm incision was made along the sciatic bone on the posterior side of the leg, exposing the sciatic nerve by gently separating the surrounding muscle tissue. A double-hooked electrode (KD-BHE, Kodou Brain Machine Technology Co. Ltd.) was used to carefully hook the sciatic nerve, ensuring that there was no contact between the double-hooked nerve and the adjacent muscle tissue. A dual-channel electrical stimulator (Hangzhou Nuanxinjia Electronic Technology Co., Ltd) was connected to the double-hook electrodes to deliver square-wave electrical stimulation to the sciatic nerve. To ensure a secure fit of the MMA to the skin, medical tape was applied, and the MMA was tightly adhered to the skin. Standard Ag/AgCl electrodes (LT-302, Shanghai Litu Medical Devices Co., Ltd) were used as the reference and grounding electrode for all electrophysiological recordings.

### Supplementary information

Supplemental Material
