# Supplementary material for: Multichannel microneedle dry electrode patches for minimally invasive transdermal recording of electrophysiological signals
Source: Microsyst Nanoeng. 2024 May 31;10:72. doi: 10.1038/s41378-024-00702-8 (PMC11143369; doi:10.1038/s41378-024-00702-8)
Supplement: Supplementary file 1 — Supplemental Material [file 41378_2024_702_MOESM1_ESM.docx]

Supplemental Material

**Multi-channels Microneedle dry electrode patch for minimally invasive transdermal recording of electrophysiological signals**

Zhengjie Liu,^1^ Xinyuan Xu,^1^ Shuang Huang,^2^ Xinshuo Huang,^1^ Zhibo Liu,^1^ Chuanjie Yao,^1^ Mengyi He,^1^ Jiayi Chen,^1^ Hui-jiuan Chen, ^1,^* Jing Liu, ^3,^* Xi Xie^1,2,^*

^1^State Key Laboratory of Optoelectronic Materials and Technologies, School of
Electronics and Information Technology; Guangdong Province Key Laboratory
of Display Material and Technology, Sun Yat-Sen University, Guangzhou, China
^2^School of Biomedical Engineering, Sun Yat-Sen University, Guangzhou, China

^3^The First Affiliated Hospital of Sun Yat-Sen University, Guangzhou, China

* Corresponding authors, E-mail:

chenhuix5@mail.sysu.edu.cn, liuj753@mail.sysu.edu.cn, xiexi27@mail.sysu.edu.cn

**S1. The fabrication procedure of MMA**


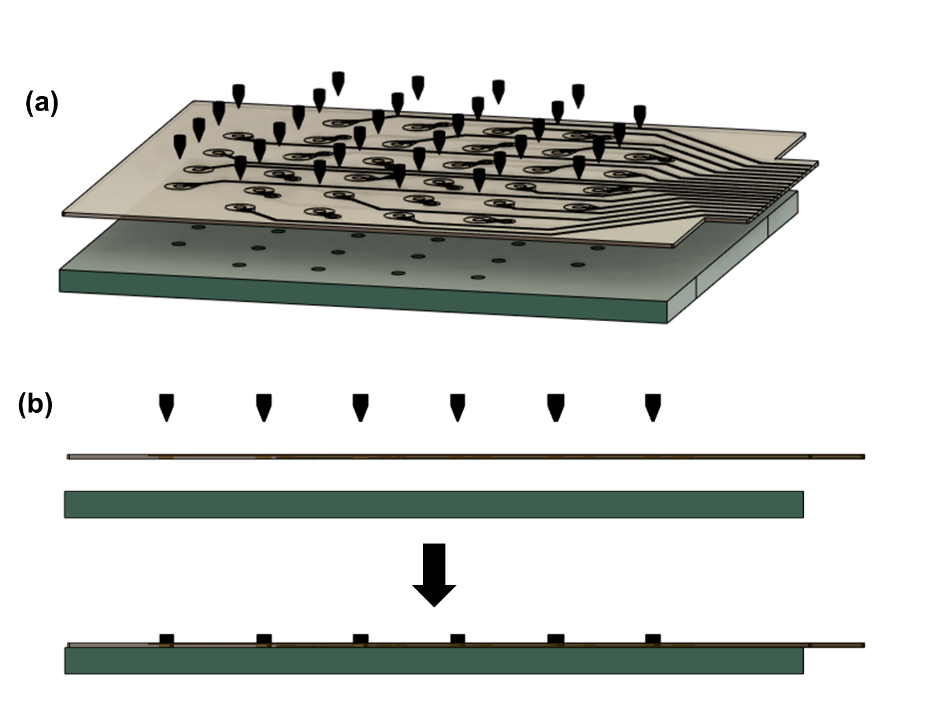


Figure S1-1. Schematic diagram of a flexible circuit board assembled with microneedles. (a) The flexible circuit board was stacked onto the FR4 substrate, ensuring precise alignment of the on the flexible circuit board with the corresponding holes in the FR4 substrate. This alignment allowed for the vertical insertion of microneedles into the pad vias of the flexible circuit board, simplifying subsequent soldering operations. (b) Thirty-two microneedles were vertically inserted into the corresponding vias of the FR4 substrate. The height at which the microneedle heads protruded from the flexible circuit board matched the thickness of the FR4 substrate.

**S2. Mechanical simulation via COMSOL Multiphysics**


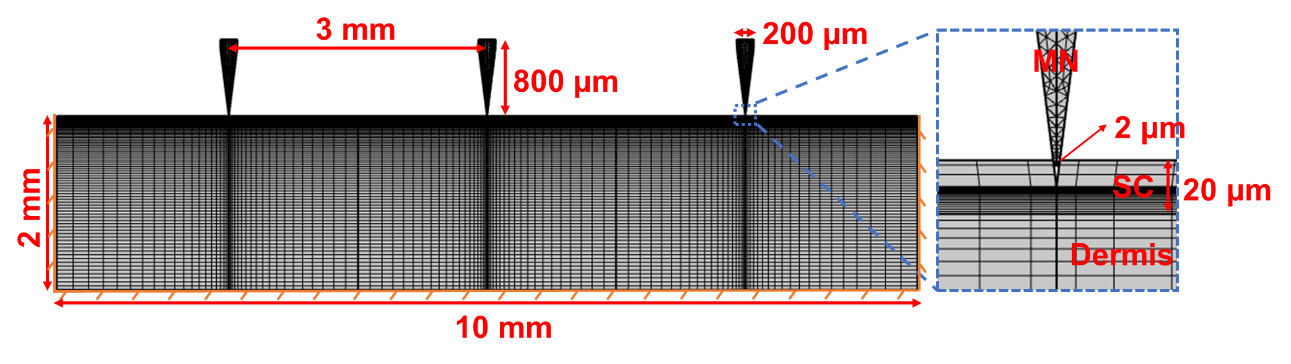


Figure S2-1. COMSOL simulation schematic and detailed dimensional parameters. The microneedles were obtained by adding rounded corners to a polygonal geometric shape. The skin was modeled as a two-layer material composed of the stratum corneum and dermis, with dimensions of 2.02×10 mm². The relevant physical parameters used in the simulation (Table S1) included 1) the Young's modulus (E_SC, E_Dermis), Poisson's ratio (ν_SC, ν_Dermis), and density (ρ_SC, ρ_Dermis) for the stratum corneum and dermis layers. 2) The skin relaxation time (τ_v). Fixed constraints were applied to the lateral and bottom edges of the skin model, and a specified displacement was applied to the microneedles in the Y-direction. The skin model utilized mapped pre-defined partitioned meshing to ensure sufficient refinement of the edge mesh in the contact area with the microneedles. The microneedles were meshed using free triangular elements.

Table S1.

Relevant physical parameters for simulation

| Symbol | Value | Definition | Reference |
| --- | --- | --- | --- |
| $E_{SC}$ | 34 kPa | Young's modulus of Stratum corneum | 1 |
| $E_{Dermis}$ | 3.4 MPa | Young's modulus of dermis | 1 |
| $\nu_{SC}$ | 0.48 | Poisson's ratio of Stratum corneum | 2 |
| $\nu_{Dermis}$ | 0.48 | Poisson's ratio of dermis | 2 |
| $\rho_{SC}$ | 1300 kg/m3 | Density of Stratum corneum | 3 |
| $\rho_{Dermis}$ | 1200 kg/m3 | Density of dermis | 3 |
| $\tau_{v}$ | 180 s | Relaxation time of the skin | 1 |


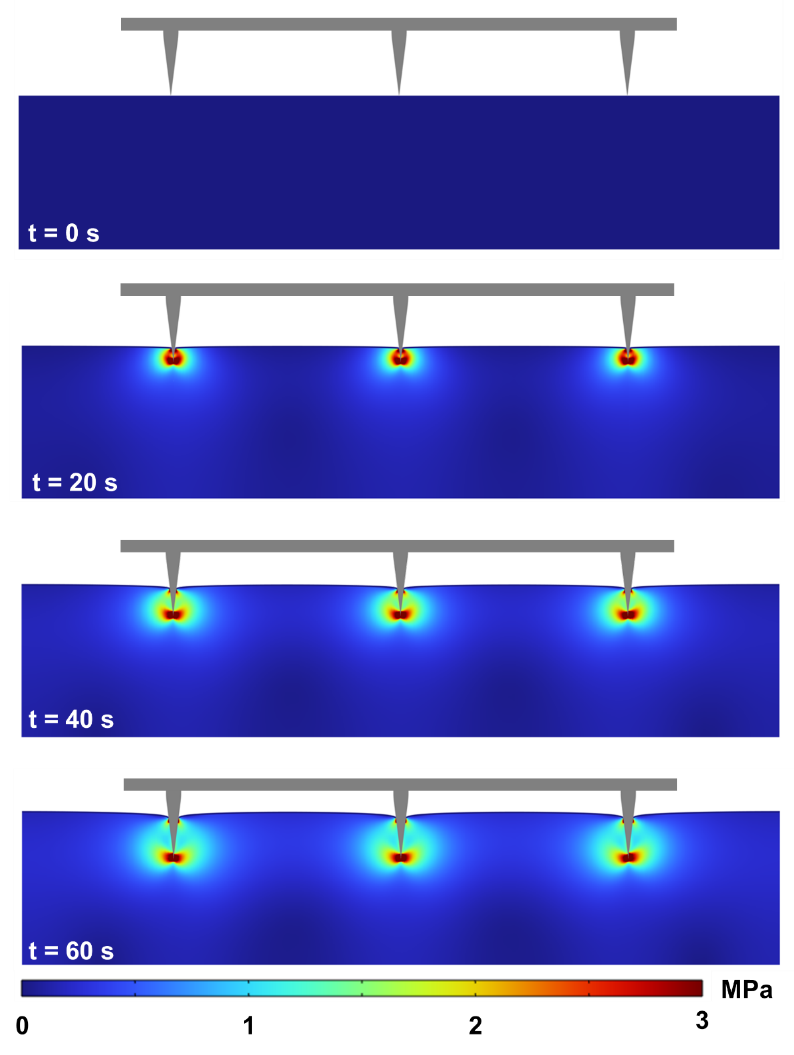


Figure S2-2. Stress distribution in the skin during insertion. The time interval of the simulation was 20 seconds.


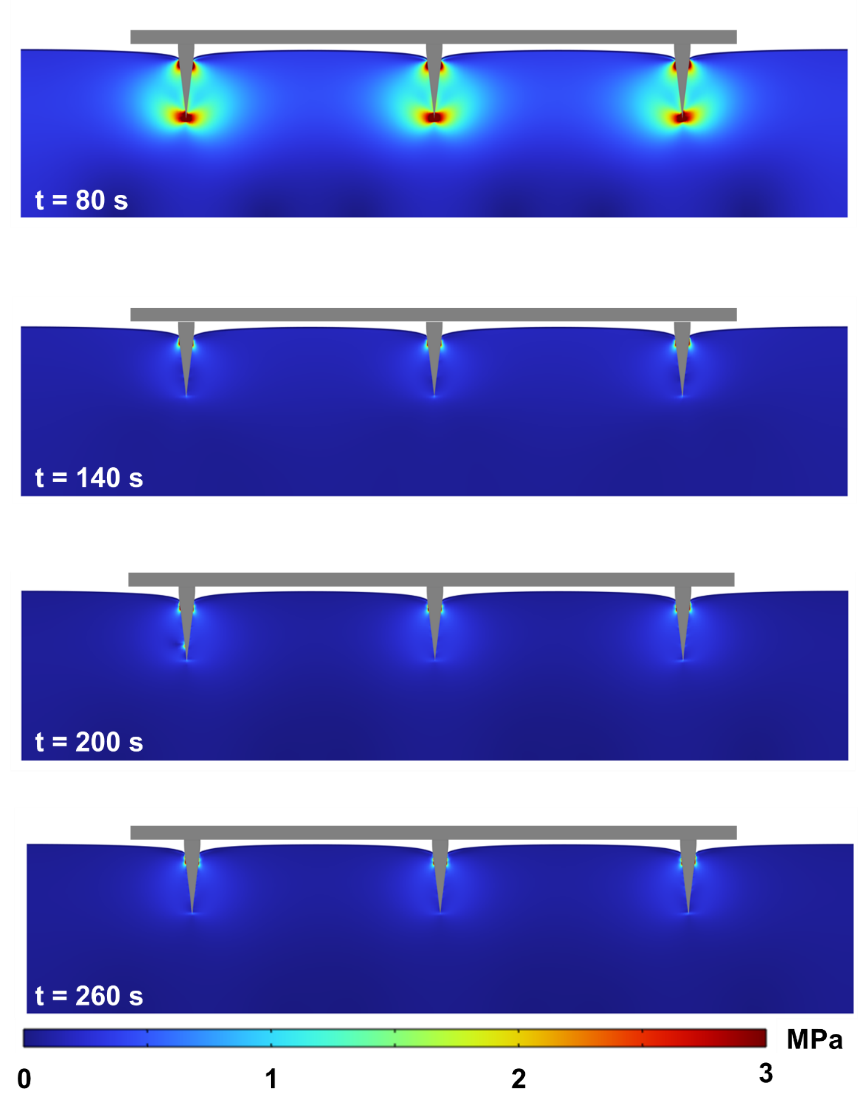


Figure S2-3. Stress distribution in the skin during resting. The time interval of the simulation was 60 seconds.


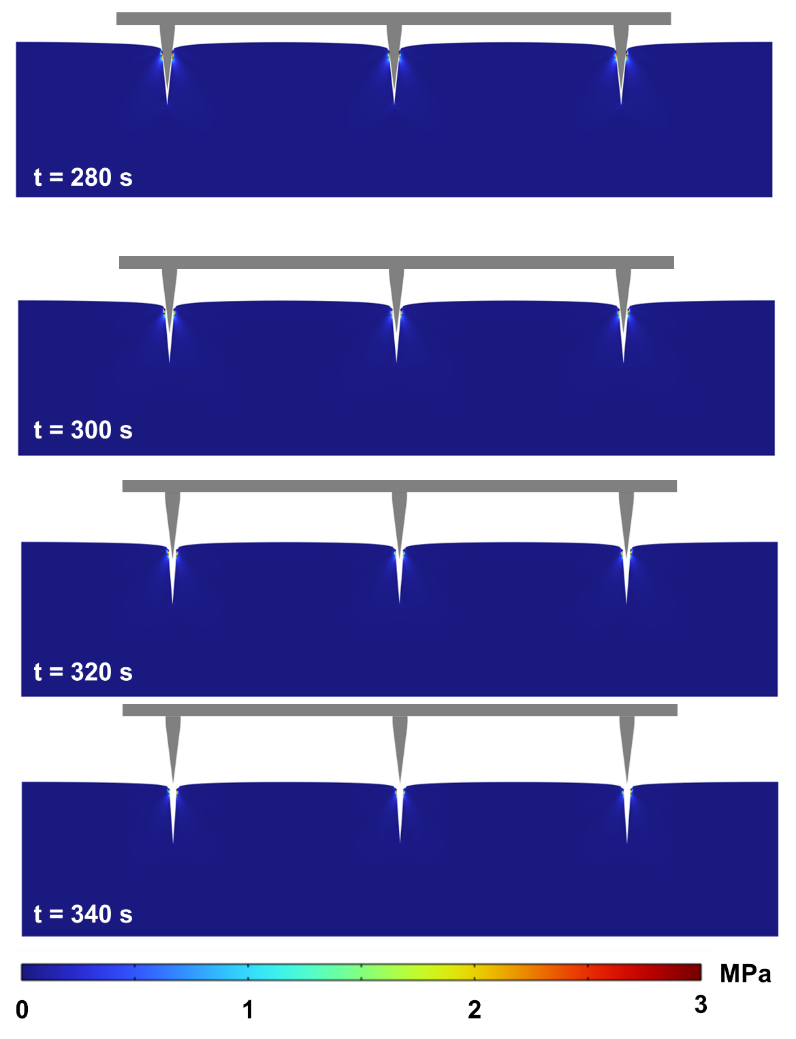


Figure S2-4. Stress distribution in the skin during removal. The time interval of the simulation was 20 seconds.

**S3. Mechanical testing of MMA**


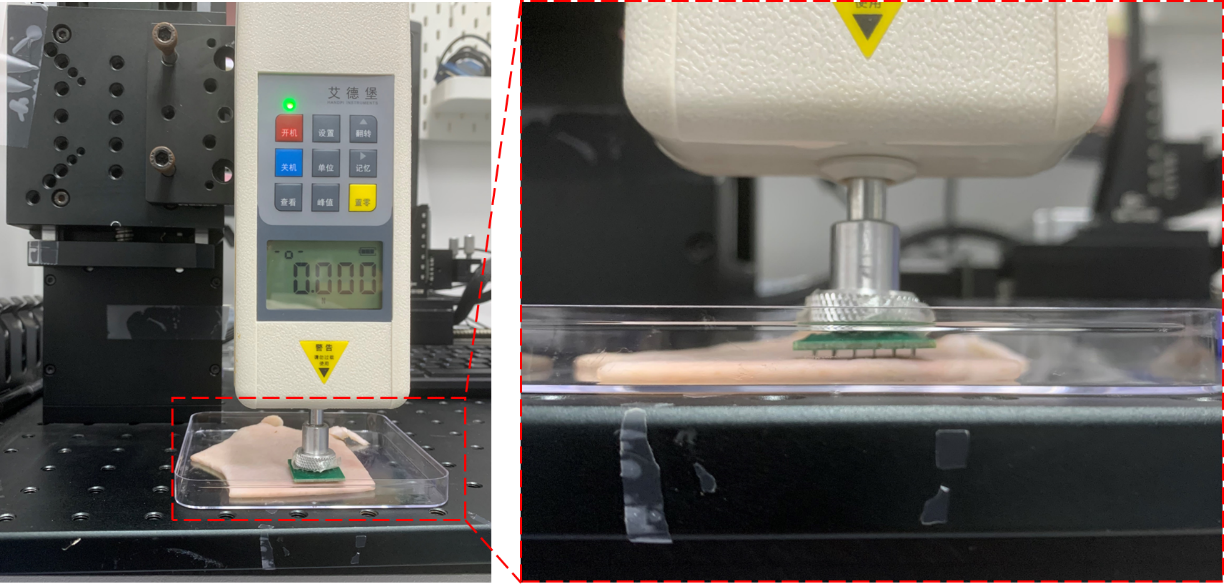


Figure S3. Diagram of a mechanical test for insertion-removal of microneedles in pigskin. The microneedle array's electrode tips were fixed downward and secured to dynamometer probes with double-sided adhesive. A motorized displacement stage controlled the y-axis movement (perpendicular to the pigskin surface), simulating the process of inserting and removing the MMA.

**S4. Impedance testing of electrodes and apples/tomatoes**


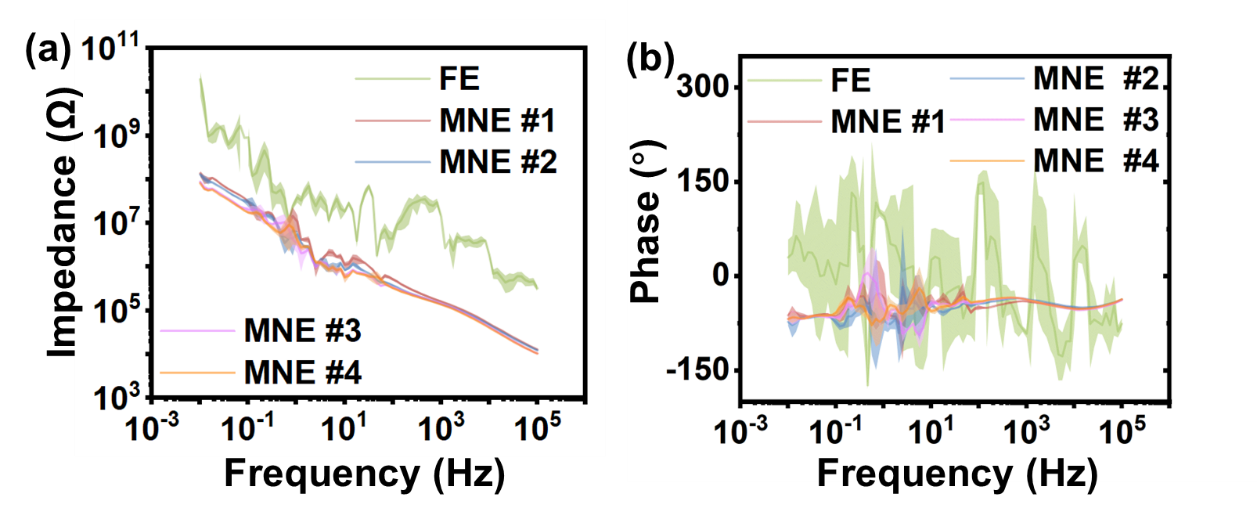


Figure S4-1. Comparison between FE and MNE for impedance measurement of apples. A pair of MNEs was inserted into the apple with an insertion depth of 1 mm and the spacing between the electrodes was set to 3 cm, and four different locations of the apple were selected for impedance detection. (a) Impedance versus frequency plot of MNE and FE detection of apple. (b) Phase versus frequency plot of microneedle and planar electrode detection of apple.


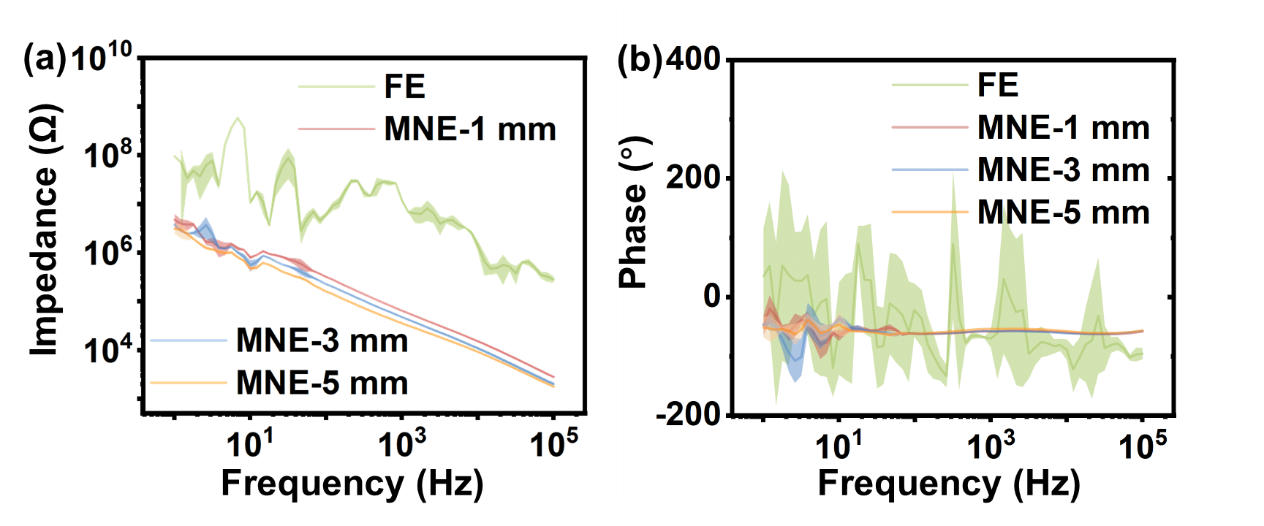


Figure S4-2. Comparison between FE and MNE for impedance measurement of tomatoes. A pair of MNEs was inserted into the tomato, and the spacing between the electrodes was set to 3 cm, and the insertion depth of the MME was adjusted for impedance detection. (a) Impedance versus frequency plot of MNE and FE detection of tomato. (b) Phase versus frequency plot of microneedle and planar electrode detection of tomato.

**S5. Electrical simulation of MNE/FE-skin model**


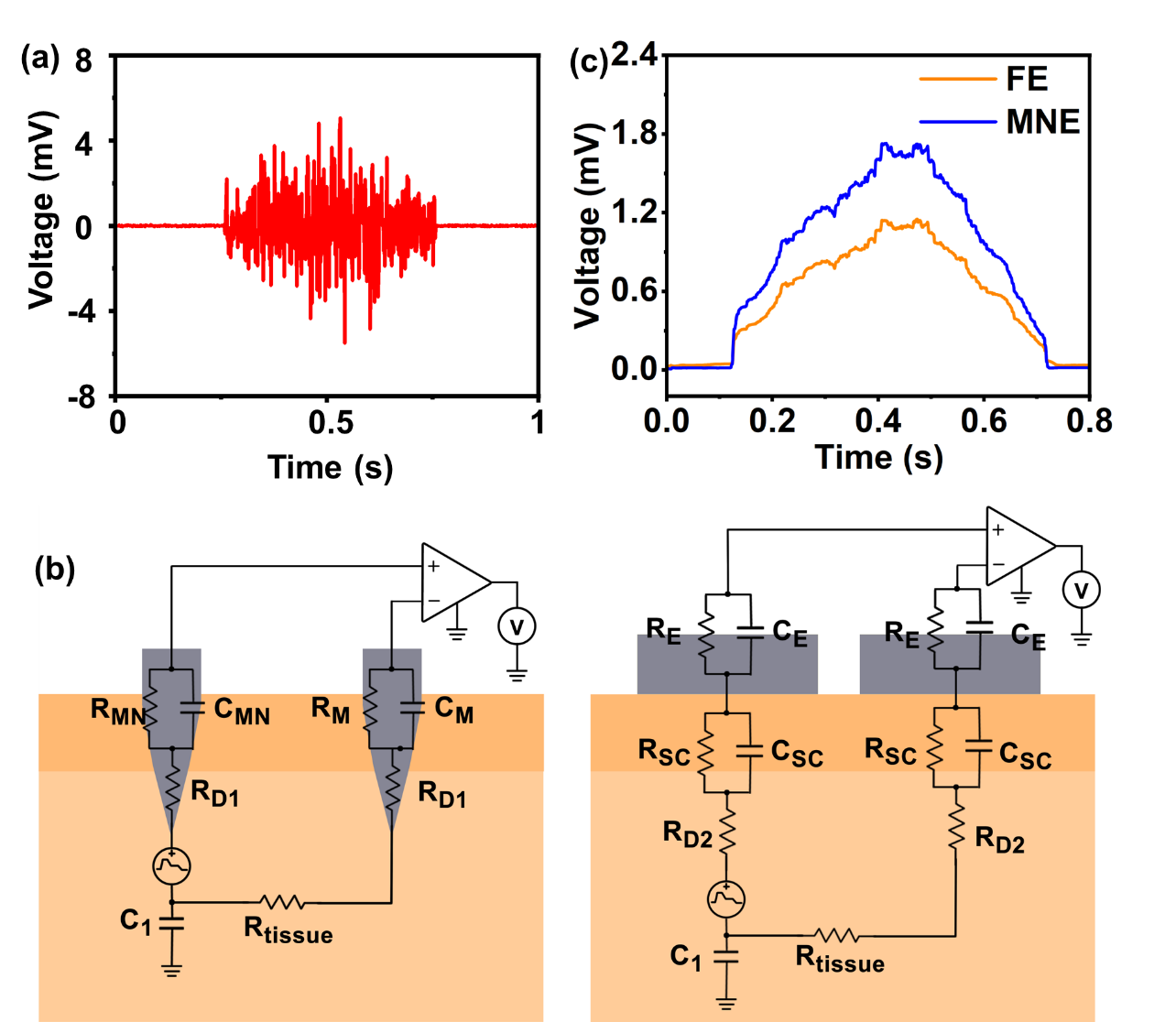


Figure S5-1. Simulation of electrophysiological signals acquired by MNE/FE. (a) Testing with a typical EMG signal. The data was imported into the PWL voltage element. (b) Schematic of the complete equivalent circuit model for electrophysiological testing. In this case, the PWL voltage element was used to simulate the generation of electrophysiological signals. Since the detection of electrophysiological signals was achieved by measuring the potential difference between two electrode points, an instrumentation amplifier AD620 was used to detect the potential difference between two electrodes, and an oscilloscope was used to detect the output of the instrumentation amplifier. (c) RMS amplitude of EMG signal detected by the MNE/FE.


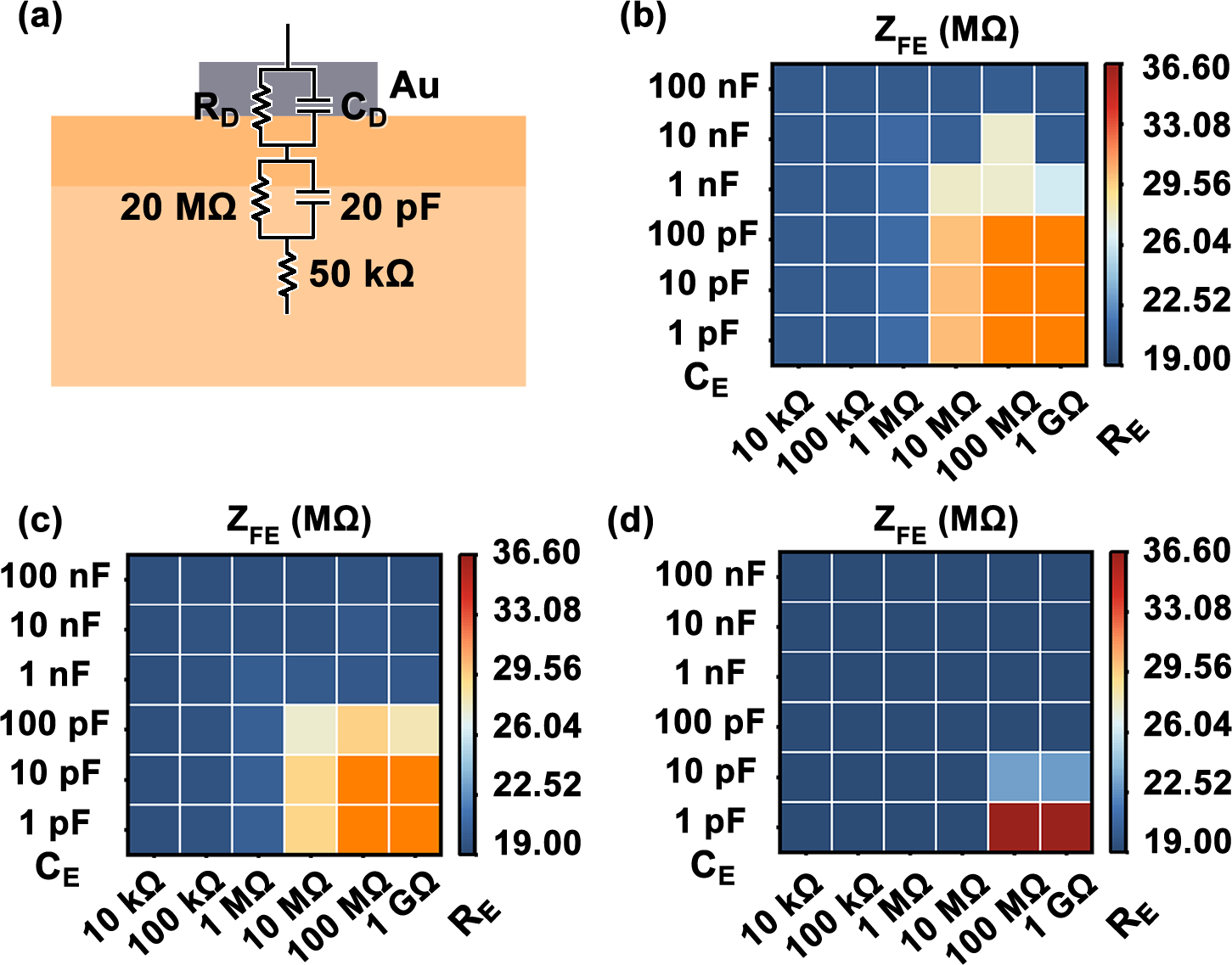


Figure S5-2. Impedance simulation of FE model. The values of $R_{E}$ and $C_{E}$ were varied to simulate different contact states and the total FE-skin impedance was calculated by Multisim software. (a) Schematic diagram of FE model. (b) The heat map summarized the $Z_{FE}$ calculations conducted at a frequency of 1 Hz across varying $R_{E}$ and $C_{E}$ values. (c) The heat map summarized the $Z_{FE}$ calculations conducted at a frequency of 10 Hz across varying $R_{E}$ and $C_{E}$ values. (d）The heat map summarized the $Z_{FE}$ calculations conducted at a frequency of 1000 Hz across varying $R_{E}$ and $C_{E}$ values.

**S6. Pictures of MMA and MESAS**


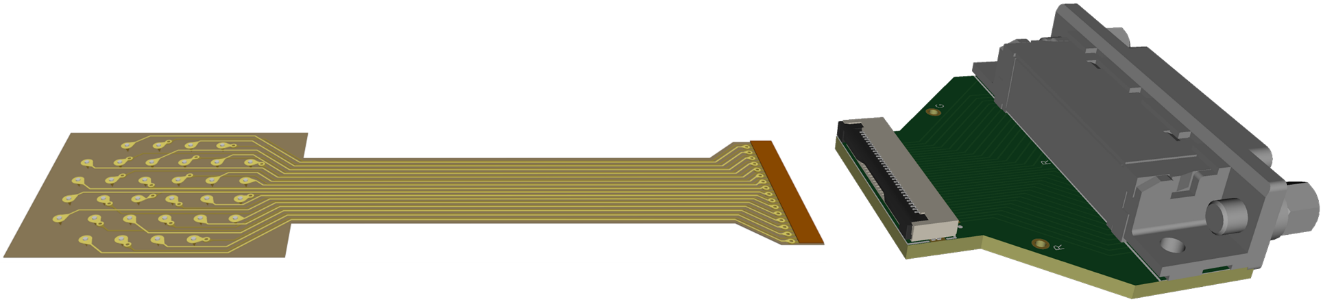


Figure S6-1. Diagram of MMA and adapter connector.


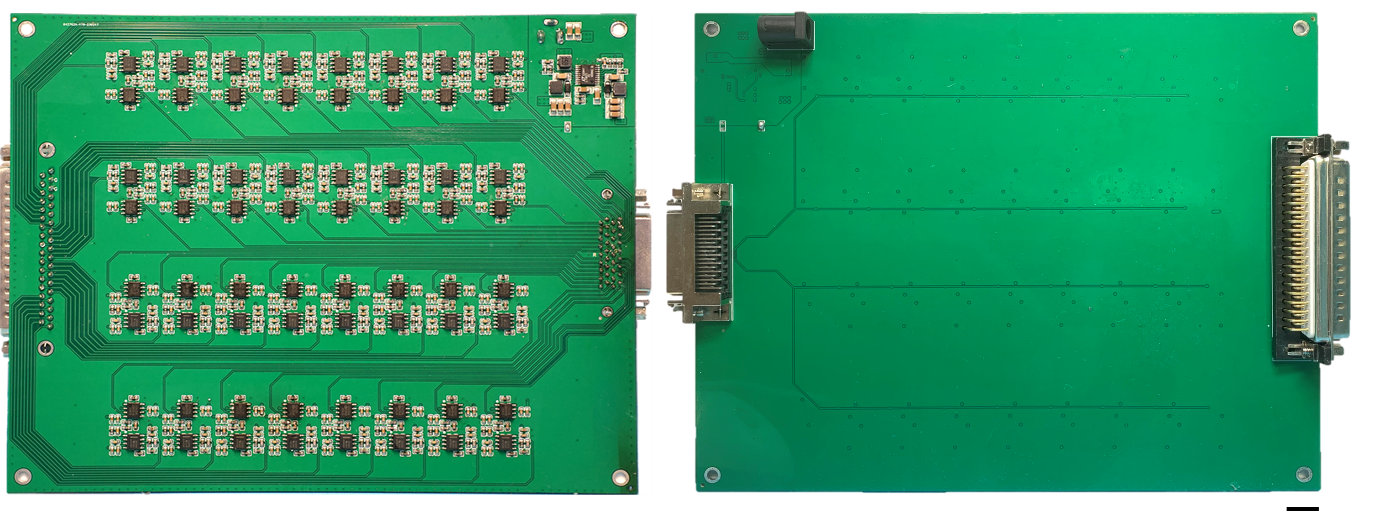


Figure S6-2. Real image of the PCB. (Front and back, scale 1 cm)
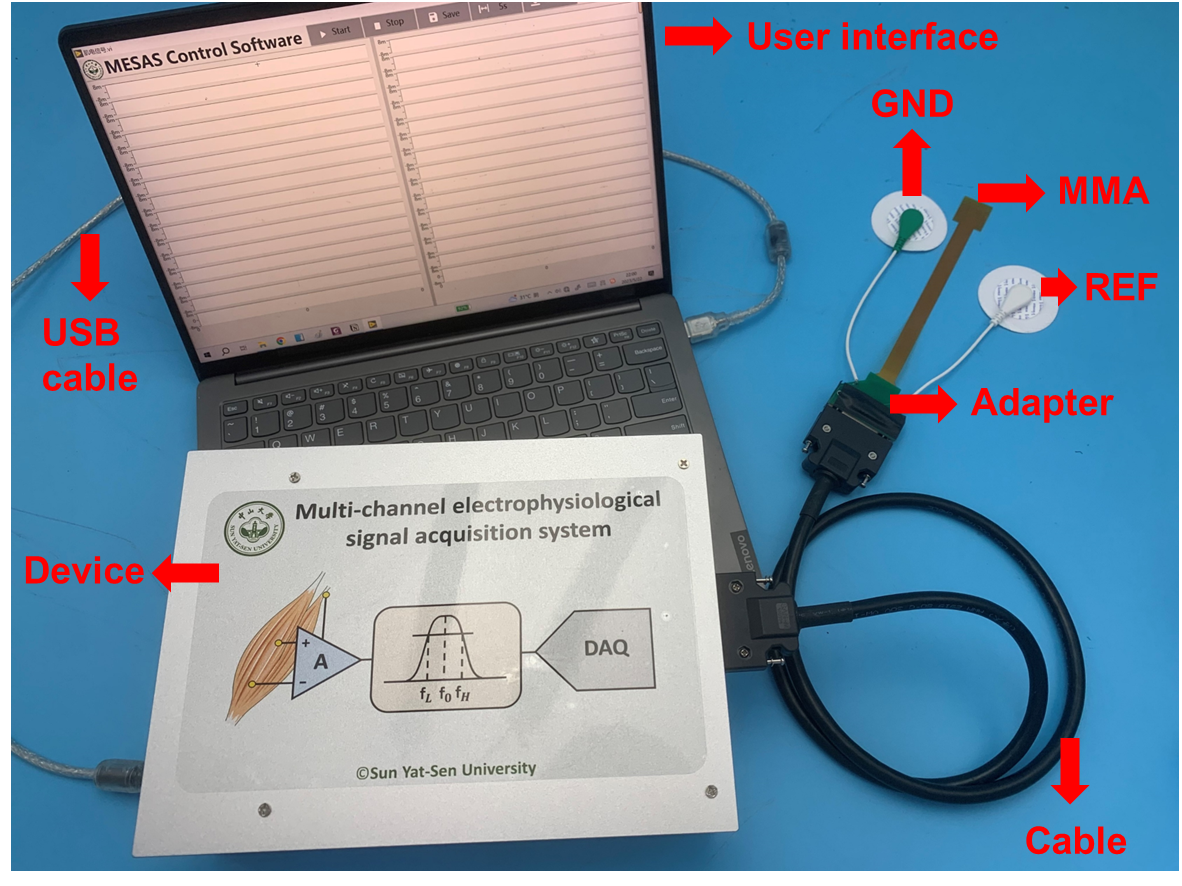


Figure S6-3. Photograph of the MESAS. In order to reduce the interference of external electromagnetic fields on electrophysiological detection, a shielded box wrapping device is prepared using aluminum alloy material, while a shielded wire connecting device with an adapter plate is used.


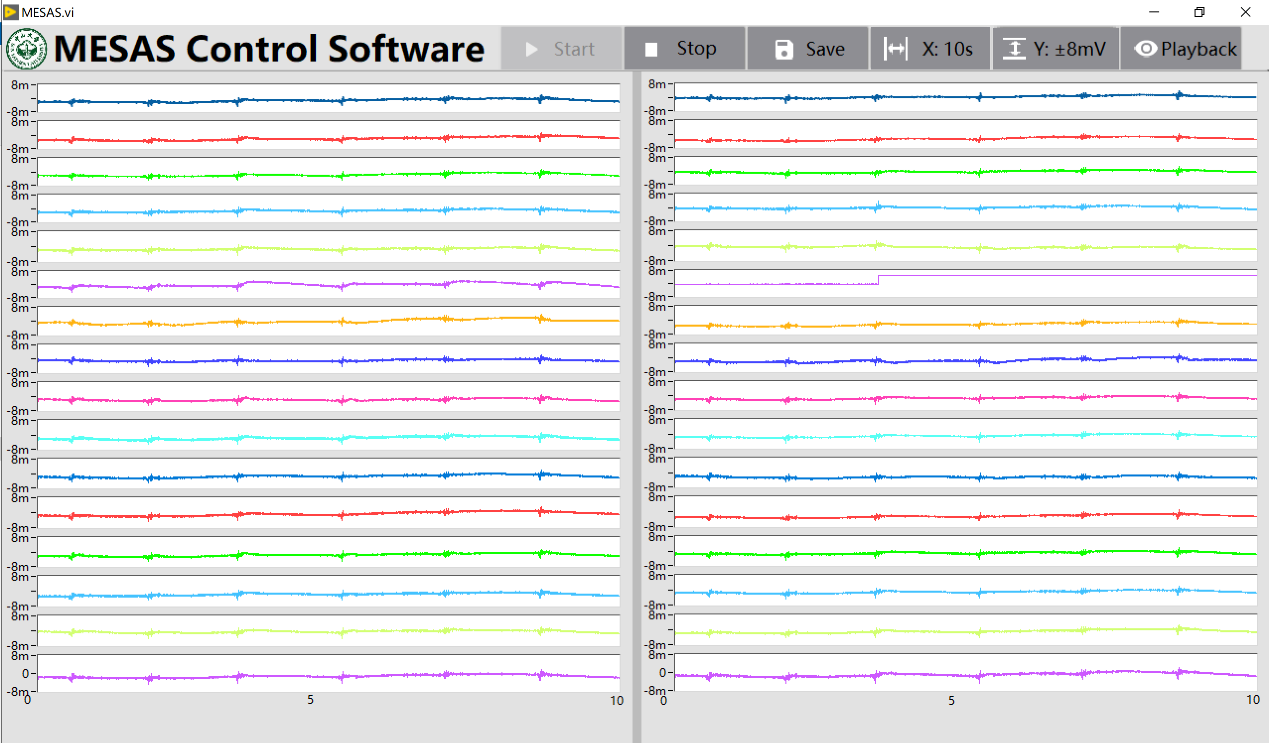


Figure S6-4. Multi-channel electrophysiology acquisition software user interface. The user interface comprises of two distinct sections: the waveform graph display and the control panel. The latter houses buttons for recording electrophysiological signals, such as 'Start', 'Stop', 'Save', 'X-axis range', 'Y-axis range' and 'Playback'. The 'Start' and 'Stop' buttons allow users to initiate and terminate signal recordings, while selecting the 'Save' button stores the multi-channel electrophysiological signal data to a TDMS file. Clicking the 'Stop' button, on the other hand, halts the recording and prevents the electrophysiological signal data from being saved. The 'X-axis Range' and 'Y-axis Range' buttons are employed to determine the horizontal and vertical scales of the waveform diagram. The 'Playback' button imports electrophysiological signal data that has been received beforehand into the software and displays the signals on the waveform diagram, thereby playing back recorded data. This button enables the user to review previous recordings.

Table S2. System specifications

| Number of channels | 32 |
| --- | --- |
| Acquisition frequency | 100 SPS ~ 50 kSPS |
| Bandwidth | - 1. Hz ~10 kHz |
| Detection voltage range | -5 mV~5 mV |
| Resolution | 152 μV |
| Input impedance | 10 GΩ |

**S7. Surface morphology of the punctured skin**


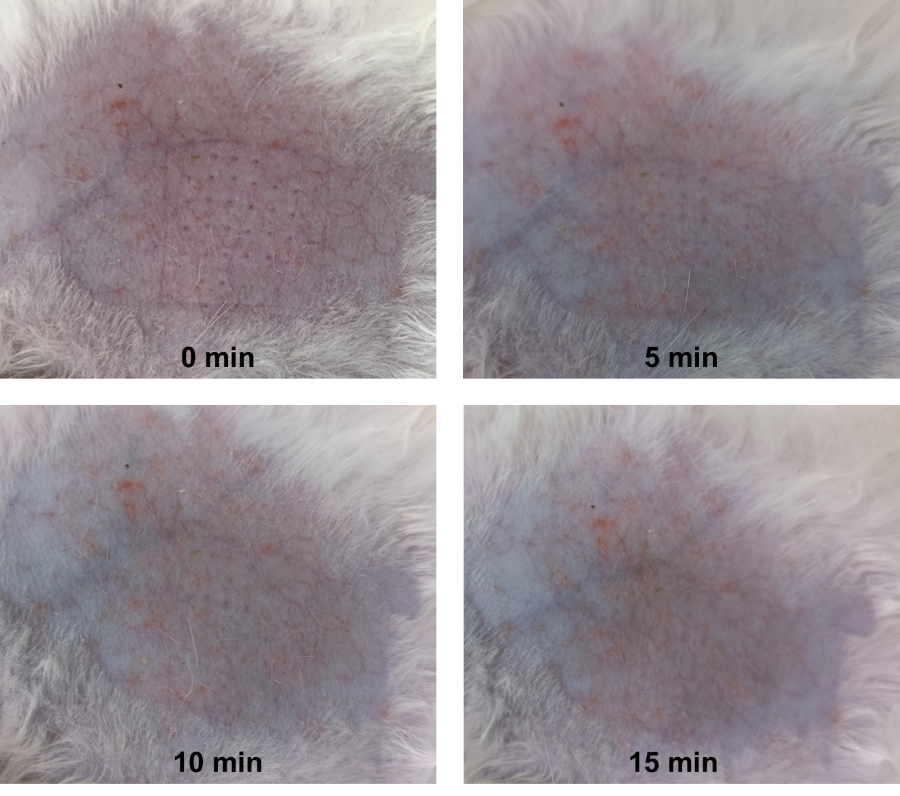


Figure S7. The MMA was applied onto the skin of the rabbit with a single press. After removal of the MMA, the natural recovery of the skin began. The surface morphology of the punctured skin was recorded every 5 minutes.

**S8.** **Distribution map of electrodes**


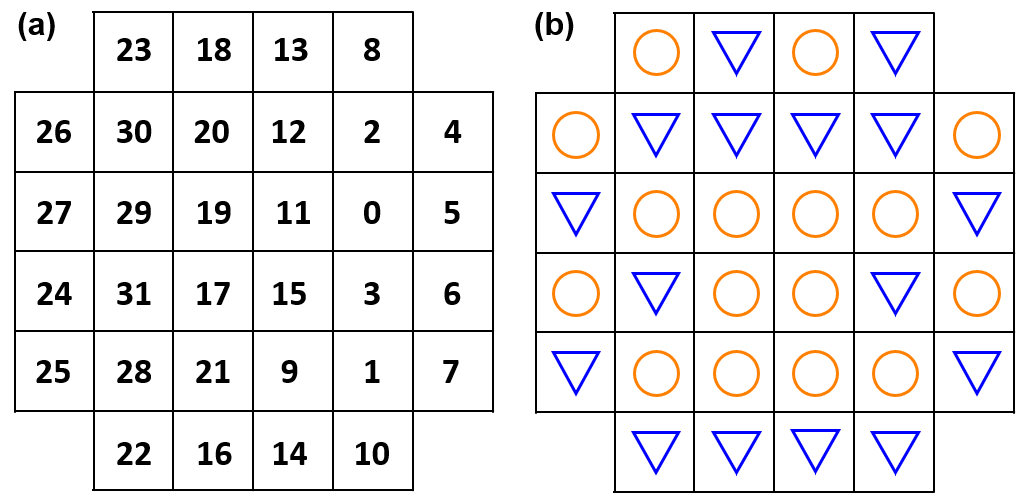


Figure S8. Auxiliary grid distribution for electrode placement. (a) The auxiliary grid distribution was employed to locate the 32-channel MNEs, with the numbers corresponding to the recorded channels. (b) Electrode distribution of the device with integrated FEs and MNEs. The blue triangles represented the respective positions of the MNEs, while the orange circles represented the corresponding positions of the FEs.

**S9. Signals recorded by FEs and MNEs.**


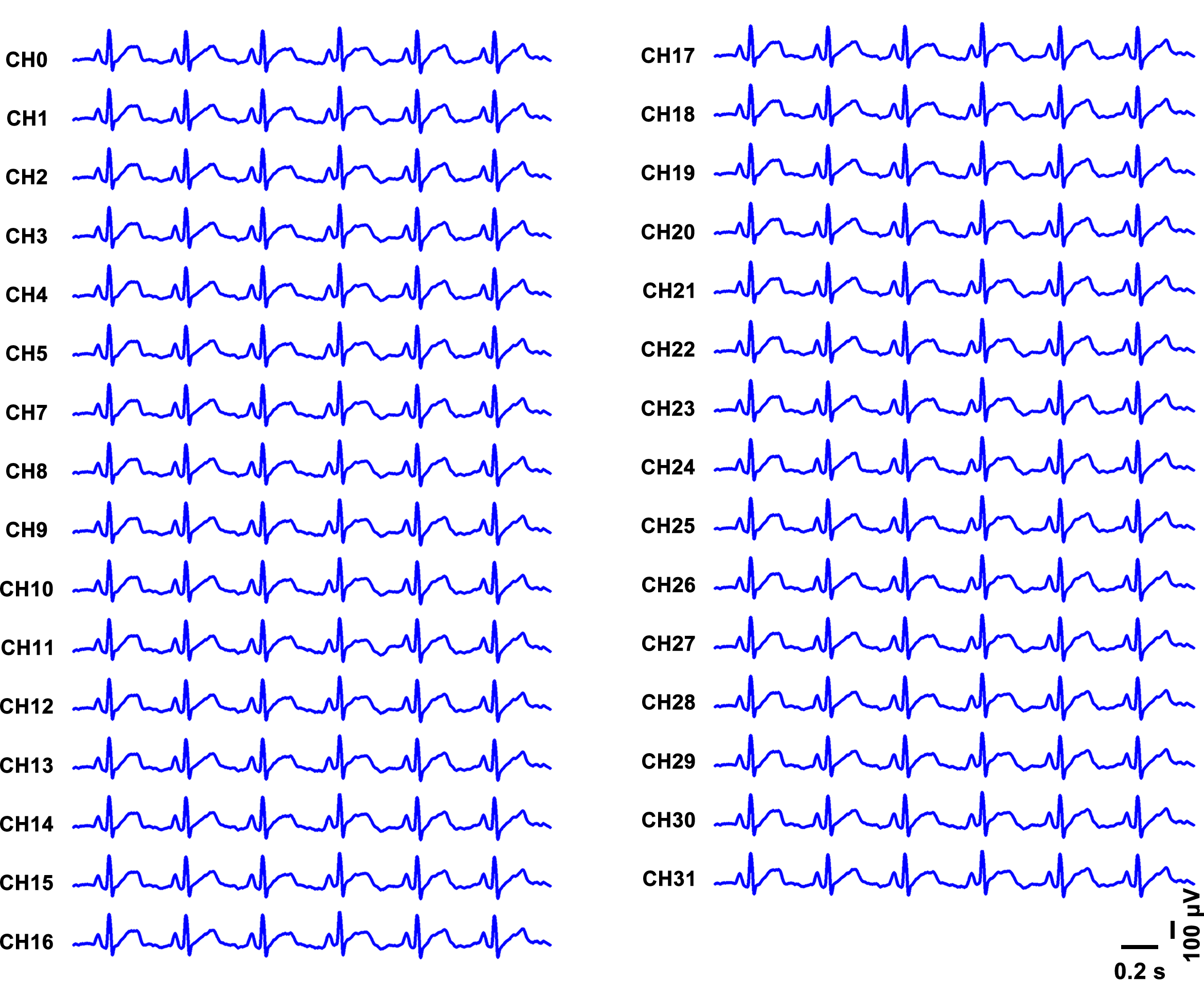


Figure S9-1. Segments of recorded ECG signals from all MNEs.


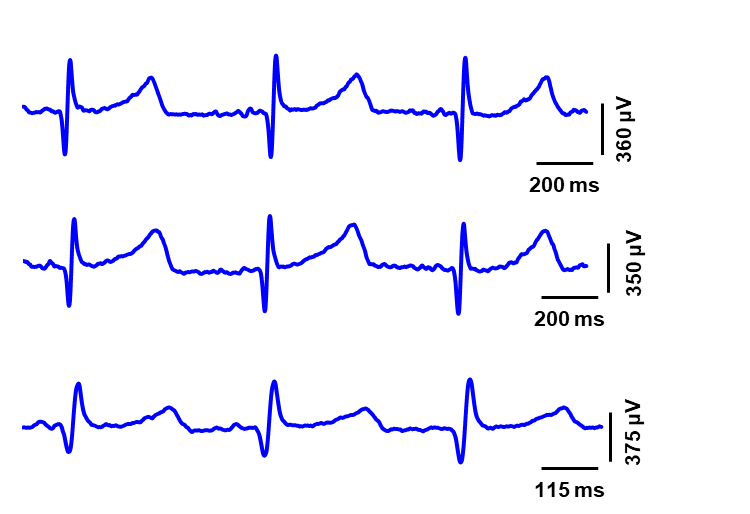


Figure S9-2. Measurement of ECG signals every 15 minutes.


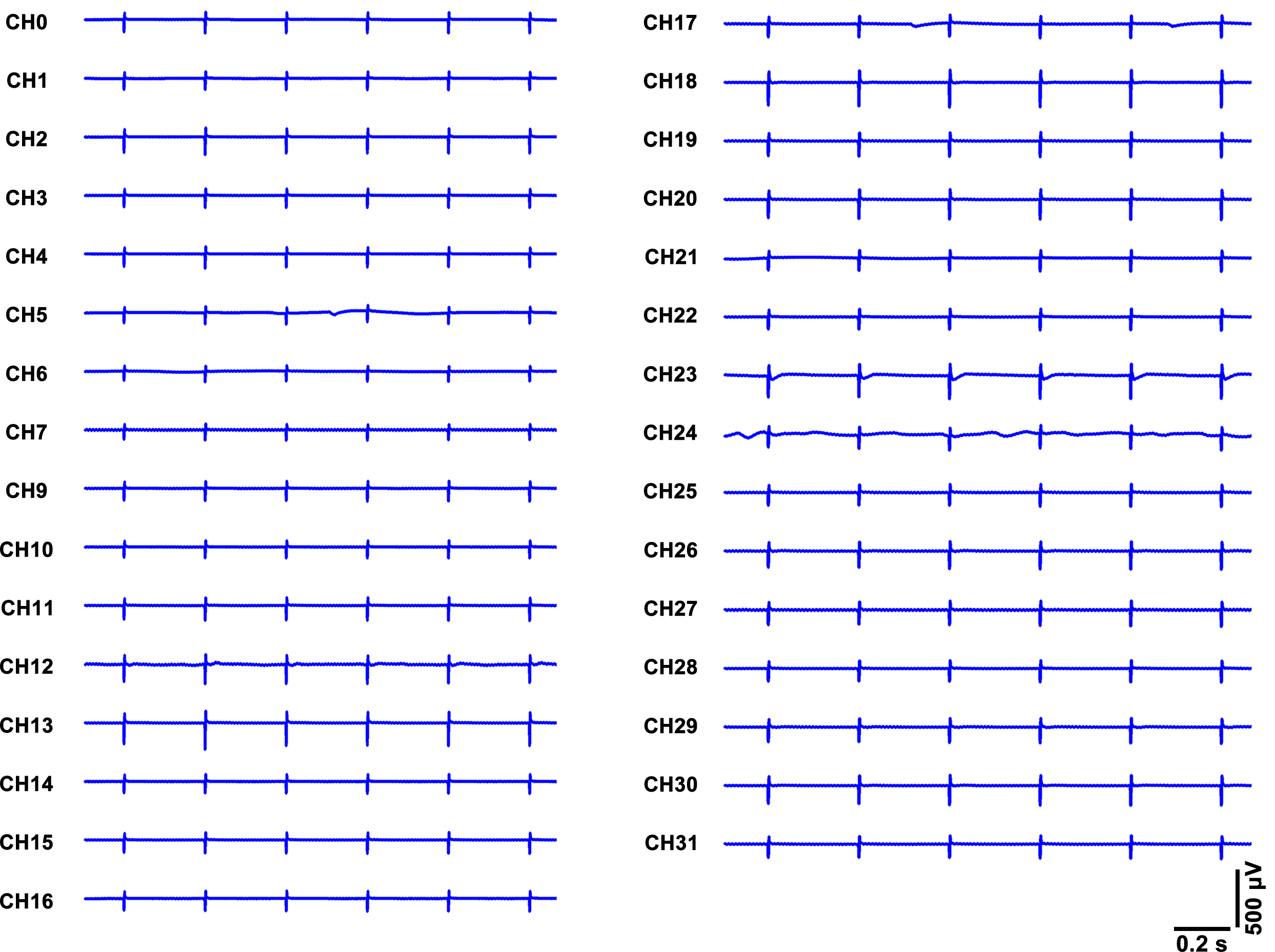


Figure S9-3. Segments of recorded EMG signals from all MNEs.


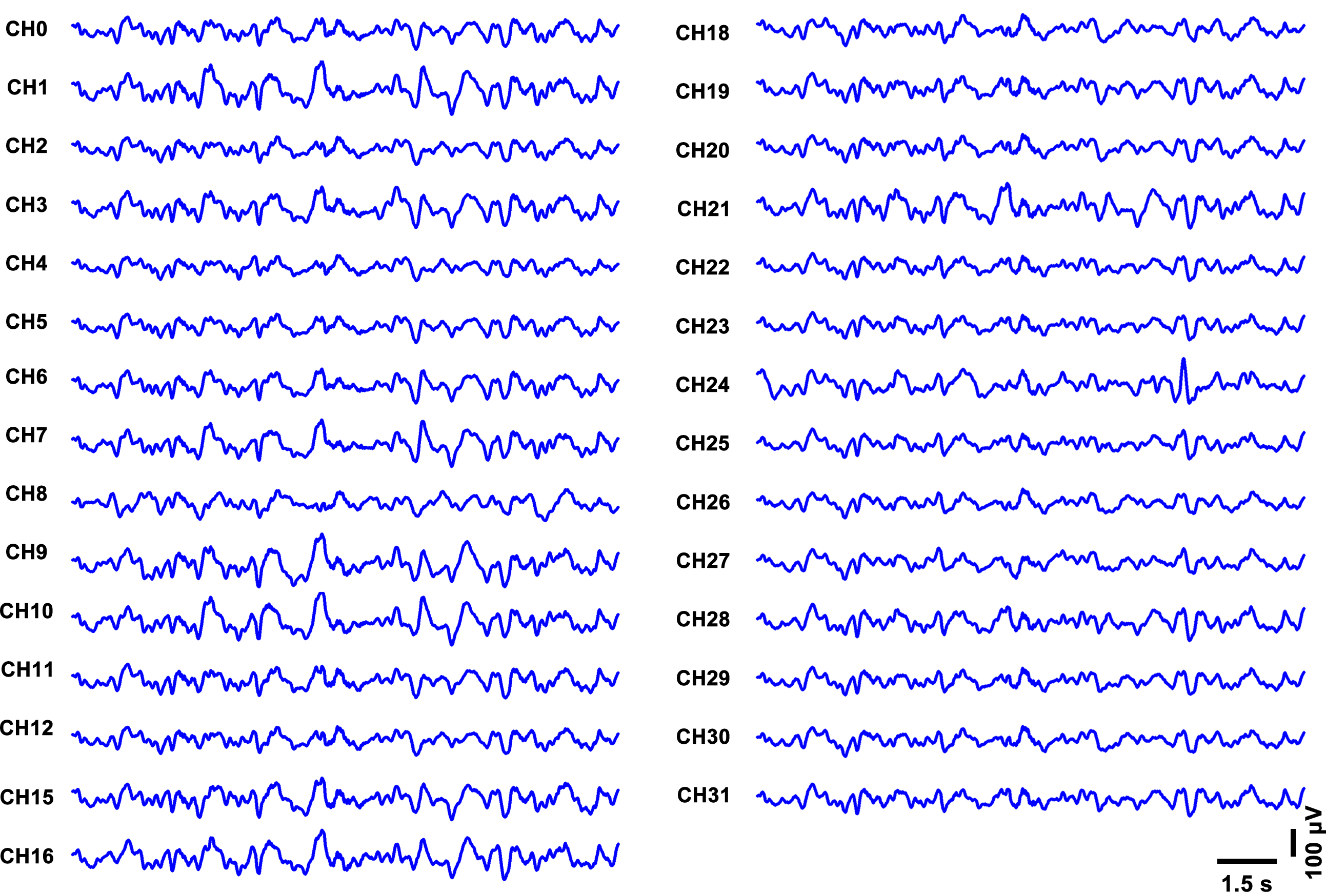


Figure S9-4. Segments of recorded EEG signals from all MNEs.


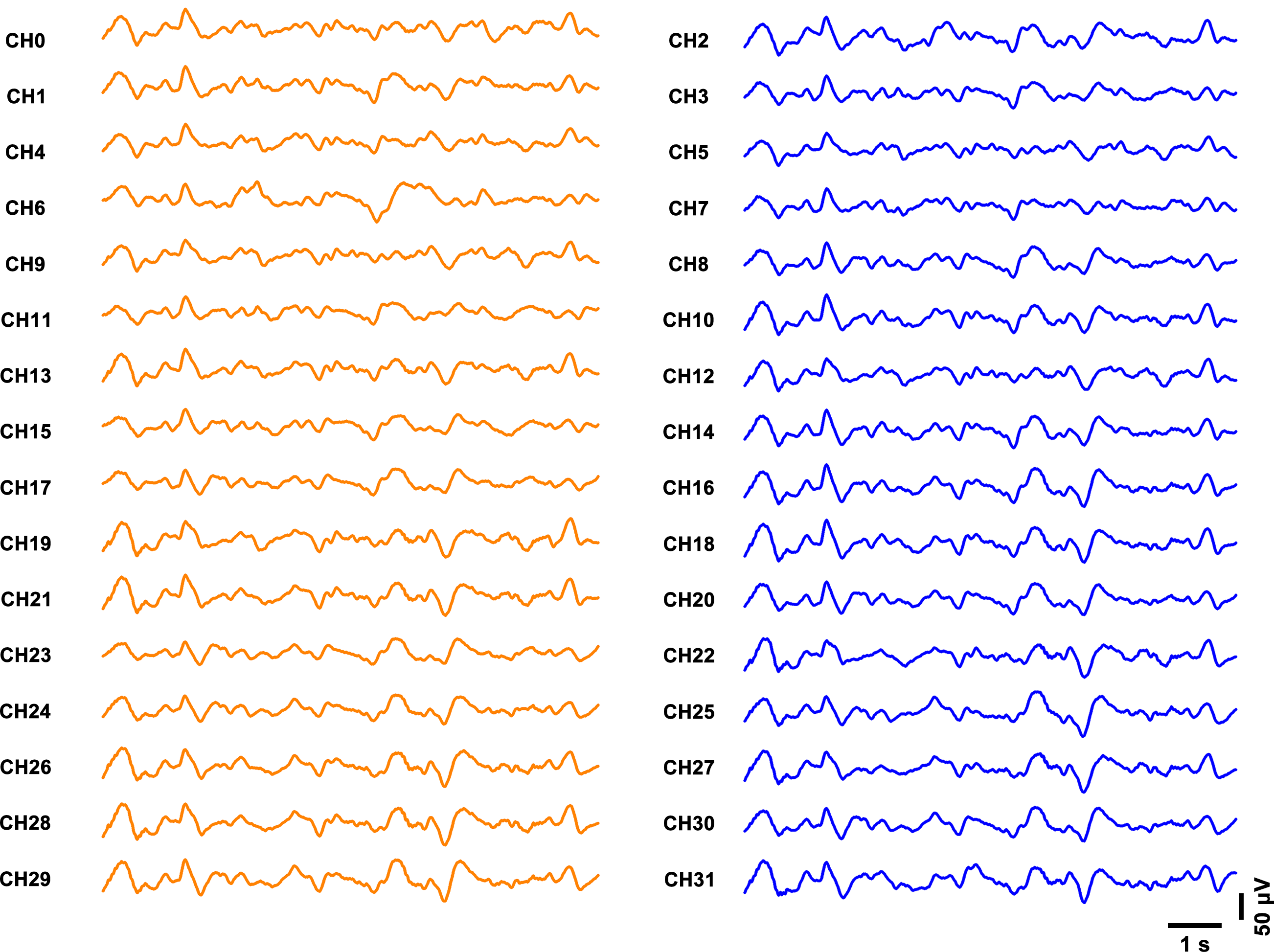


Figure S9-5. Segments of EEG signals were recorded separately for FEs and MNEs.

**References:**

1. https://pubs.acs.org/doi/10.1021/acsami.8b09563#

2. https://www.sciencedirect.com/science/article/pii/S0021929005002162

3. https://doi.org/10.1115/IMECE2013-64443
